# Mitochondrial AK3 inhibits nuclear β-catenin localization and its activation through enhancing mitochondrial activity

**DOI:** 10.1038/s41419-026-08777-z

**Published:** 2026-04-22

**Authors:** Muhah Jeong, Shin-Hyeon Ryu, Young-Sin Cho, Do-Hyeong Na, Jongyeon Baek, Jihoon Nah, Ki Woo Kim, Yong-Keun Jung

**Affiliations:** 1 School of Biological Sciences, Seoul National University, Seoul, South Korea; 2 Interdisciplinary Graduate Program in Genetic Engineering, Seoul National University, Seoul, South Korea; 3 Department of Biochemistry, Chungbuk National University, Cheongju, South Korea; 4 Departments of Oral Biology and Applied Life Science, BK21 FOUR, Yonsei University College of Dentistry, Seoul, South Korea

**Keywords:** Energy metabolism, Cell signalling, Cancer metabolism

## Abstract

The aberrant Wnt/β-catenin signaling is tightly associated with developmental disorders and tumorigenesis. However, spatial regulation of cytoplasmic β-catenin with regard to its nuclear accumulation and signaling activation remains poorly understood. Herein, we show that mitochondrial adenylate kinase 3 (AK3), which is involved in the TCA cycle, regulates nuclear β-catenin localization and its activation. Transcriptome profiling across multiple cancer patient datasets revealed that AK3 and oxidative phosphorylation pathway are highly correlated with Wnt/β-catenin signaling and prognosis of patients. Using cancer cell lines, we found that AK3 enzymatic activity inhibited β-catenin signaling and cell proliferation by attenuating nuclear β-catenin accumulation. Intriguingly, mitofusins (MFN1 & 2) were identified as β-catenin interactors and demanded for the AK3-mediated β-catenin signaling regulation. Additionally, β-catenin-mitofusins interactions were enhanced by AK3 expression but disrupted by treatment with CCCP. These results suggest that metabolically active mitochondria induced by AK3 restrain β-catenin signaling through modulating the β-catenin-mitofusins interactions.

## Introduction

Emerging evidence is demonstrating that mitochondria are crucial for various aspects of cancer progression by regulating metabolism and cell signaling [1]. Metabolic suppression of mitochondrial oxidative phosphorylation (OxPhos) and enhanced glycolytic pathway are prevalent in many cancer cells, a phenomenon called ‘Warburg effect’ [2]. Nevertheless, since cancer cells inevitably depend on mitochondria for energy-demanding processes, the absolute rate of ATP production through OxPhos still surpasses that of glycolysis [3]. Furthermore, mitochondrial deregulation results in the conditions favoring cancer cell survival; mitochondria-originated reactive oxygen species (ROS) induce genomic instability, hyperactivate oncogenic signaling cascades, and provide immunosuppressive tumor microenvironment (TME) and metastatic potential [4–6].

Wnt/β-catenin signaling is pivotal to embryo development and adult tissue homeostasis, and its dysregulated activation leads to various diseases, including cancer [7]. Upon Wnt activation, β-catenin is released from β-catenin destruction complex into the cytoplasm, stabilized, and imported into nucleus to activate the signaling [8]. However, the spatial regulation of β-catenin in the cytoplasm regarding its nuclear translocation remains largely unclear, especially its interactions with subcellular organelles. At the plasma membrane, formation of cadherin-catenin complex not only mediates adherens junction, but restrains the β-catenin signaling in nucleus [9]. The interaction between β-catenin signaling and mitochondria is controversial. While several reports suggest that mitochondrial retrograde signaling activates β-catenin signaling depending on mitochondrial activity [10–13], other studies demonstrate that enhancing mitochondrial pyruvate metabolism inhibits Wnt/β-catenin signaling in tumor cells [14, 15]. The β-catenin has also been detected in mitochondrial fraction even though its physiological implications are unknown yet [16, 17].

Adenylate kinases (AKs) are integral enzymes for cellular energy homeostasis, catalyzing the reversible transfer of a phosphate group between adenosine and guanosine nucleotides, in distinct intracellular localization [18]. Since mitochondria utilize mitochondrial AKs; AK2 in intermembrane space and AK3 and AK4 in the matrix, to support OxPhos or to regulate stress response [19–21]. AK2 deficiency leads to mitochondrial impairment and aberrant ROS production, resulting in metabolic diseases and severe combined immunodeficiency [22–24]. Although increasing studies have reported the association of mitochondrial AK2 and 4 with cancer progression and cell death [21, 25–28], little is known about AK3 except for its enzymatic function in the tricarboxylic acid (TCA) cycle which is a central mitochondrial pathway producing energy carriers to drive ATP generation, where it recycles GTP [20, 29].

In this study, we provide evidence that AK3 regulates β-catenin localization to suppress its signaling by promoting mitochondrial activity. By analyzing multiple transcriptomic datasets of cancer patients and performing in vitro mechanistic studies, we propose an association among AK3, OxPhos, and β-catenin signaling.

## Results

### Down-regulation of AK3 in tumors is associated with Wnt/β-catenin signaling and oxidative phosphorylation

**Fig. 1:**
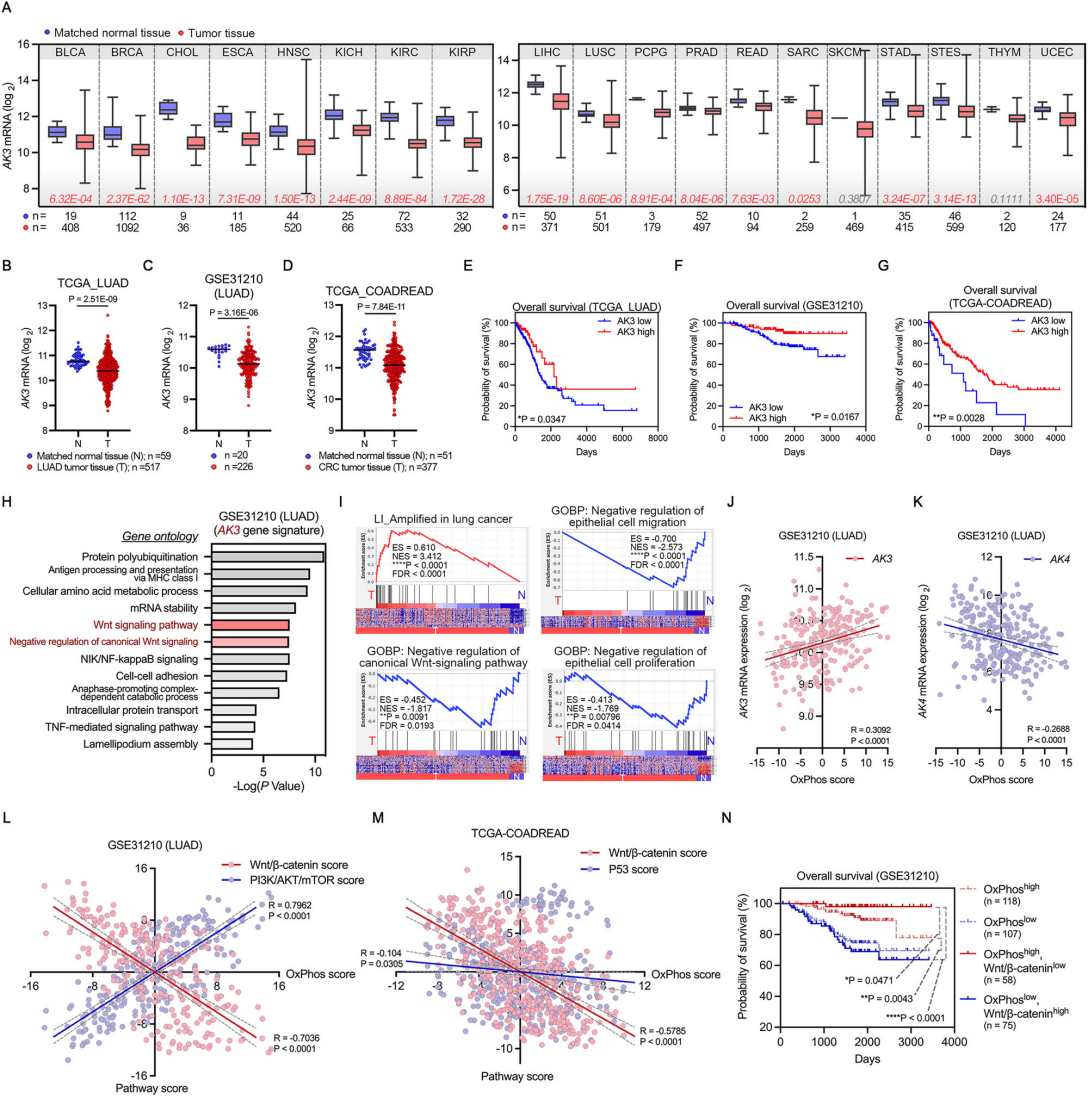
AK3 is negatively correlated to the poor prognosis of LUAD and CRC patients, Wnt/β-catenin signling, and OxPhos pathways. **A** AK3 mRNA expression in TCGA microarray datasets. The cancer types showing downregulated expression of AK3 in tumors were gathered and statistically analyzed; bladder urothelial carcinoma (BLCA), breast invasive carcinoma (BRCA), cholangiocarcinoma (CHOL), esophageal carcinoma (ESCA), head and neck squamous cell carcinoma (HNSC), kidney chromophobe (KICH), kidney renal clear cell carcinoma (KIRC), kidney renal papillary cell carcinoma (KIRP), liver hepatocellular carcinoma (LIHC), lung squamous cell carcinoma (LUSC), pheochromocytoma and paraganglioma (PCPG), prostate adenocarcinoma (PRAD), rectum adenocarcinoma (READ), sarcoma (SARC), skin cutaneous melanoma (SKCM), stomach adenocarcinoma (STAD), stomach and esophageal carcinoma (STES), thymoma (THYM), uterine corpus endometrial carcinoma (UCEC). Microarray datasets were analyzed for AK3 mRNA expression between tumor and matched non-tumor tissues. Box-and-whisker plots were statistically analyzed by two-tailed unpaired Student’s t-test. *P* values are described below the plots (values in red, *P* < 0.05; in gray, *P* > 0.05). **B–D** Comparison of AK3 expression in LUAD and CRC patient samples. Cancer patient datasets containing microarray data, including TCGA_LUAD (B), GSE31210 (C), and TCGA_COADREAD (**D**), were analyzed to compare AK3 mRNA expression between tumor and matched non-tumor tissues. Dot plots with mean values (black bar) were statistically analyzed by paired Student’s t-test. **E–G** Overall survival analysis of patients stratified according to the AK3 expression. LUAD datasets, including TCGA_LUAD (**E**) and GSE31210 (**F**), and a CRC dataset, including TCGA_COADREAD (**G**), were statistically analyzed using log-rank test. TCGA_LUAD (*n* = 347, 82; AK3-low, -high), GSE31210 (*n* = 140, 86; AK3-low, -high) and TCGA_COADREAD (*n* = 31, 343; AK3-low, -high). **H** Gene ontology analysis of AK3 gene signature was performed and presented as a bar graph with *P* values (Fisher exact test). **I** Gene set enrichment analysis (GSEA) of AK3 gene signature. Genes were ranked by their fold changes between two phenotypes; tumor (T) and non-tumor (N). Gene sets with *P* < 0.05 and FDR < 0.25 were considered significantly enriched. Heatmap of the gene sets is shown at the bottom of the GSEA plots. **J**,**K** AK3 and AK4 mRNA levels with OxPhos scores in LUAD. Pearson correlation coefficients and *P* values between OxPhos scores and AK3 (**J**) or AK4 (**K**) mRNA expressions were statistically analyzed in GSE31210. Linear regression lines along with 95% confidence bands (gray) were shown. Methods section describes the procedure for calculating the OxPhos score. **L**,**M** Correlations between OxPhos and Wnt/β-catenin, PI3K/AKT/mTOR, and P53 pathways in LUAD and CRC tumors and non-tumors. Pathway scores were calculated and Pearson correlation coefficients were statistically assessed in GSE31210 (**L**) and TCGA_COADREAD (**M**). Linear regression lines along with 95% confidence bands (gray) were shown. **N** Kaplan–Meier survival curves of patients from GSE31210 classified by OxPhos and Wnt/β-catenin pathway scores. Statistical significance was analyzed using log-rank test. (*P < 0.05, **P < 0.01, ***P < 0.001, ****P < 0.0001).

To understand the role of AK3, we assessed the mRNA expression in The Cancer Genome Atlas (TCGA) Pan-Cancer datasets (*n* = 7988 tumor samples, *n* = 751 matched non-tumor samples of 22 cancer types) (Fig. 1A, B, D and Fig. S1D). *AK3* expression was broadly downregulated in the tumor tissues across 22 cancer types compared to the normal adjacent tissue. Among the cancers, we further investigated lung adenocarcinoma (LUAD) and colorectal carcinoma (CRC) which are considered to be strongly associated with Wnt/β-catenin signaling pathway [30–32]. From the analysis of LUAD and CRC patient datasets containing microarray data (LUAD; *n* = 332 tumor samples, *n* = 125 matched non-tumor samples; CRC; *n* = 212 tumor samples, *n* = 194 matched non-tumor samples), we observed significant downregulation of *AK3* in both tumor tissues (Fig. 1B–D and Fig. S1A, B, D–H). The downregulation of *AK3* was observed at every stage of LUAD tumor (Fig. S1C). Patients harboring *AK3*^low^ tumors also show poor survival in both LUAD and CRC patients (Fig. 1E–G). These data imply that *AK3* might be a tumor-suppressor gene.

We next set the ‘*AK3* gene signature’ to identify the metabolic or signaling pathways that are associated with the role of AK3. By analyzing the LUAD dataset (GSE31210) in Fig. 1C, 2511 probes were annotated as *AK3* gene signature (Fig. S1I). The genes in *AK3* gene signature were enriched for the Wnt-signaling pathway related gene ontology terms and the NIK–NF-κB pathway was also identified as AK3-associated, whose crosstalk with mitochondria has been already reported to promote cancer progression via TME interactions [33] (Fig. 1H). To investigate an unknown mechanistic link to Wnt signaling, we performed additional analyses regarding to the canonical Wnt pathway. Gene Set Enrichment Analysis (GSEA) of *AK3* gene signature also revealed that the genes enriched for poor prognosis of lung cancer, are negatively correlated with *AK3*, whereas the genes enriched for good prognosis of cancer and negative regulation of the canonical Wnt-signaling pathway are positively correlated with *AK3* (Fig. 1I).

Since AK3 is a mitochondrial matrix enzyme involved in TCA cycle, we hypothesized that AK3 alters mitochondrial metabolism to regulate the Wnt/β-catenin signaling. We thus analyzed the expression of oxidative phosphorylation (OxPhos) genes, which were collectively summarized into a single OxPhos score [34]. As expected, we found a positive correlation between *AK3* expression and OxPhos score in both LUAD and CRC patients (Fig. 1J and Fig. S1J). On the other hand, AK4 in the mitochondrial matrix did not show such a positive correlation (Fig. 1K and Fig. S1K). Analysis of signaling pathway scores revealed that the Wnt/β-catenin pathway score, but not PI3K/AKT/mTOR and P53 pathways, showed a strong and negative correlation with OxPhos score in both LUAD (R = −0.7036) and CRC (R = −0.5785) patients (Fig. 1L, M). Stratification of the LUAD patients (GSE31210) into two groups by OxPhos scores showed that the patients with OxPhos^high^ showed higher survival rate than the patients with OxPhos^low^ (Fig. 1N). Subdividing the patients by Wnt/β-catenin scores further unveiled that the patients with OxPhos^high^: Wnt/β-catenin^low^ showed the highest survival rate, but the patients with OxPhos^low^: Wnt/β-catenin^high^ showed the lowest survival rate (Fig. 1N), indicating the significance of OxPhos and Wnt/β-catenin pathways in LUAD prognosis. Overall, AK3 is a potential candidate of tumor-suppressor and associated with mitochondrial metabolism and Wnt/β-catenin pathway.

### AK3 suppresses the β-catenin signaling and cell proliferation

**Fig. 2:**
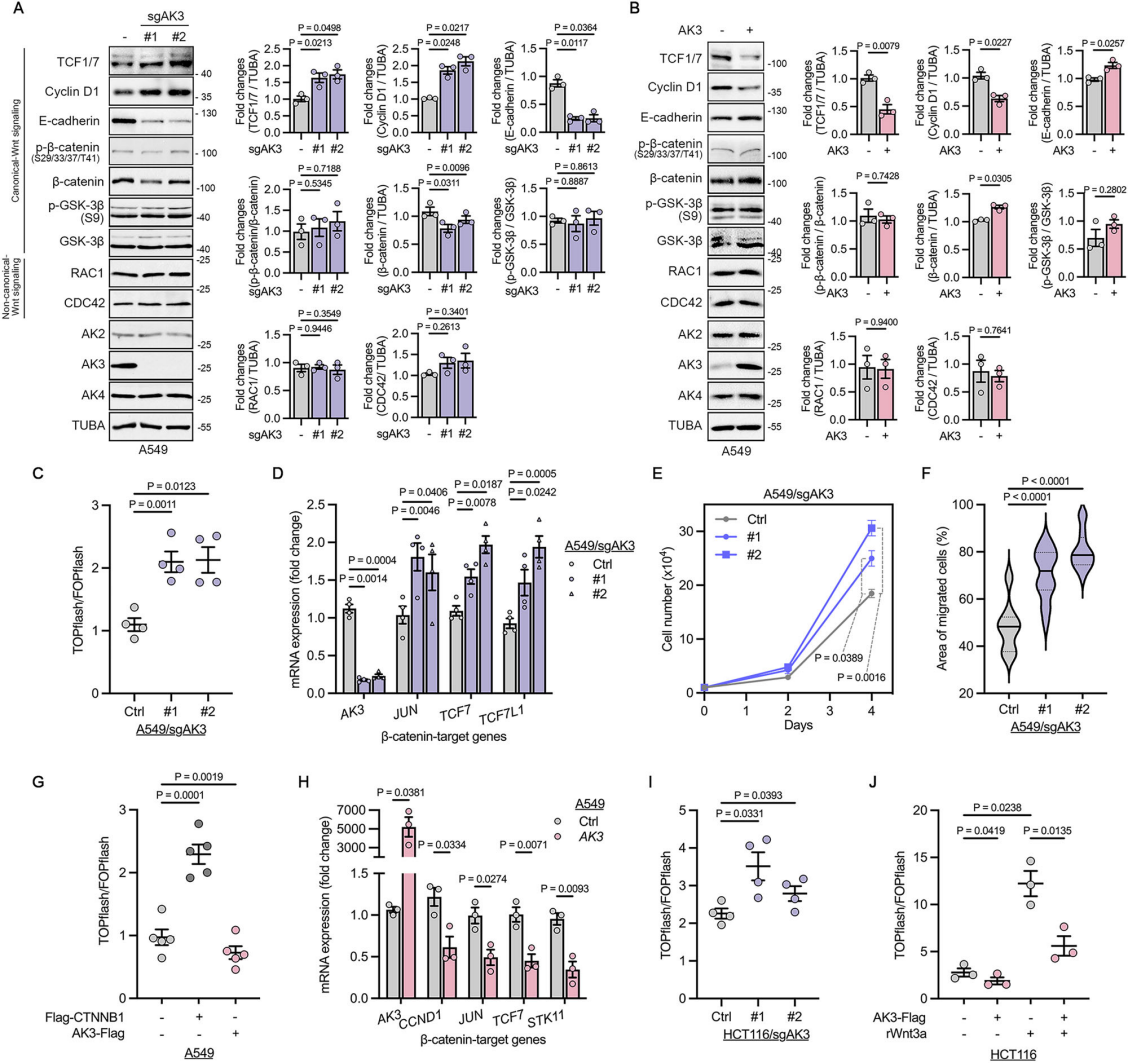
β-catenin signaling and cell proliferation are suppressed by AK3. **A**,**B** Immunoblot analysis of proteins involved in canonical or non-canonical Wnt signaling pathway in A549 AK3-knockout (KO) cells (**A**) and A549 cells expressing exogenous AK3 (**B**). AK3 KO cells were generated by two different guide RNAs (#1 or #2). Signals on the blots were quantified and statistically analyzed. *n* = 3. (**C**,**D**) Activation of β-catenin signaling in A549 AK3-KO cells assessed by TOPflash/FOPflash (**C**) and RT-PCR (**D**) assays. *n* = 4 (**C**) and *n* = 4 (**D**). (**E**) Cell proliferation rates of A549 AK3-KO cells analyzed by counting cell numbers for 4 days. *n* = 3. **F** Cell migration rates of A549 AK3-KO cells assessed by wound healing assay. Area of migrated cells after 24 h was quantified and analyzed. (Control, #1, #2; *n* = 14, 16, 14). (**G**,**H**) Inhibition of β-catenin signaling in A549 cells overexpressing AK3 for 24 h and assessed by TOPflash/FOPflash (**G**) and RT-PCR (**H**) assays. *n* = 5 (**G**) and *n* = 3 (**H**). **I** β-catenin signaling of HCT116 AK3-depleted cells analyzed by TOPflash/FOPflash assay. *n* = 4. **J** Activation of Wnt/β-catenin signaling assessed by TOPflash/FOPflash assay in HCT116 cells. Cells were transfected with AK3-Flag and treated with 100 ng/ml recombinant Wnt3a for 16 h. *n* = 3. Paired one-way ANOVA followed by dunnett’s multiple comparison tests (**A**,**C–G**,**I**,**J**). Two-tailed paired t-test (**B**,**H**). Bars and values represent mean ± s.e.m. (*P < 0.05, **P < 0.01, ***P < 0.001, ****P < 0.0001).

To address the functional association between AK3 and β-catenin signaling, we generated AK3-knockout (KO) cells using CRISPR/Cas9 system in A549 cells, the widely used cell line to investigate β-catenin signaling in LUAD [35]. With western blotting, we confirmed AK3 KO, but not that of other mitochondrial AK2 and AK4 in A549 cells (Fig. 2A). Compared to control cells, the protein levels of β-catenin target genes (TCF1/7 and Cyclin D1) increased in A549 AK3-KO cells along with significant alterations in EMT marker E-cadherin. On the other hand, the phosphorylation status of β-catenin residues (S29, S33, S37, T41), which are modified by GSK-3β and CK1 [36], remained unaffected by AK3. Consistently, GSK-3β phosphorylation and RAC1 and CDC42 levels which are associated with the canonical and non-canonical Wnt signaling respectively, were not affected (Fig. 2A). Ectopic expression of AK3 in A549 cells downregulated TCF1/7 and cyclin D1 levels with the increased levels of E-cadherin (Fig. 2B). We further examined whether AK3 affected β-catenin signaling using a qPCR and TOPflash/FOPflash assays [37]. In according to the previous results, both TCF/LEF activity represented by TOPflash assay and mRNA levels of β-catenin target genes, including *JUN*, *TCF7*, and *TCF7L1*, increased in A549 AK3-KO cells compared to control cells (Fig. 2C, D). Cell proliferation and migration also significantly increased by AK3-KO in A549 cells (Fig. 2E, F and Fig. S2A).

Conversely, we found that cell proliferation and cell migration decreased by exogenous expression of AK3 in A549 cells (Fig. S2B–D). TOPflash assay revealed that TCF/LEF activity was also reduced by ectopic expression of AK3 (Fig. 2G). In this assay, expression of Flag-*CTNNB1* (encoding β-catenin) was used as a positive control. Consistent to the reporter assay, expressions of β-catenin-target genes, such as *CCND1*, *JUN*, *TCF7* and *STK11*, were all suppressed by ectopic expression of AK3 in A549 cells (Fig. 2H). We also found that the β-catenin–E-cadherin interaction was significantly diminished in A549 AK3-KO cells, which implies the loss of adherens junctions and acquisition of mesenchymal-like property (Fig. S2E), supporting the wound healing assay results (Fig. 2F and Fig. S2D). In addition, we also examined the role of AK3 in β-catenin signaling and cell proliferation in HCT116 colon cancer cells broadly used for β-catenin signaling study [38]. As similarly observed in A549 cells, both β-catenin signaling and cell proliferation increased by AK3-depletion in HCT116 cells (Fig. 2I, and Fig. S2F, H, I), while these decreased by AK3 overexpression (Fig. S2G, J, K). Confirmatively, the activation of Wnt/β-catenin signaling following the treatment of Wnt3a ligand was also hampered by AK3 expression in HCT116 cells (Fig. 2J). Taken together, AK3 negatively regulates both transcriptional activity of β-catenin and cell proliferation.

### AK3 enzymatic activity attenuates β-catenin signaling by inhibiting nuclear β-catenin accumulation

**Fig. 3:**
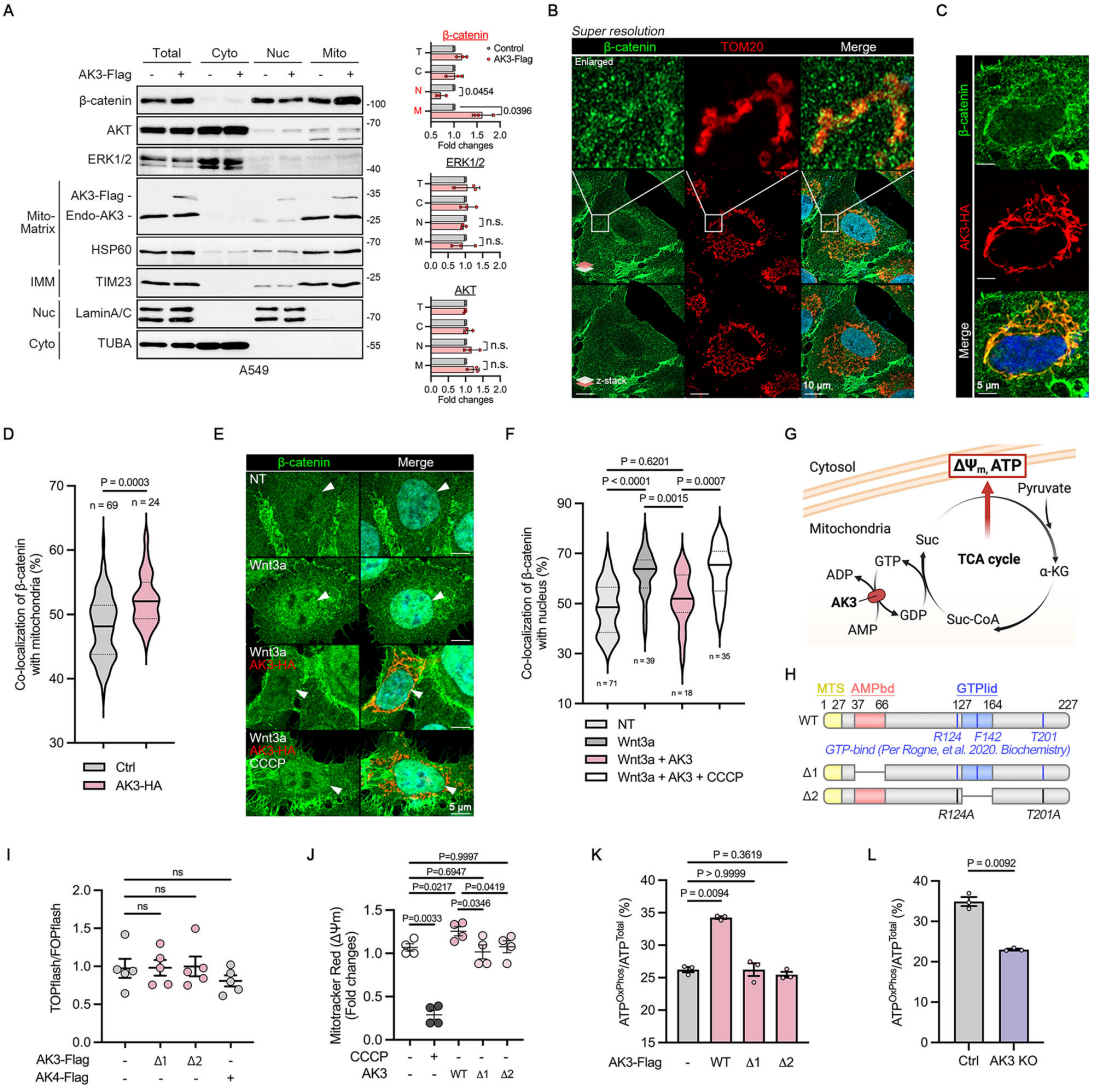
AK3 nucleotide-binding domains are required for inhibiting β-catenin signaling. **A** Subcellular fractionation assay in A549 cells overexpressing AK3-Flag for 24 h. Markers of the indicated subcellular organelles are shown (left). Nuc; nucleus, IMM; inner mitochondrial membrane, Cyto; cytosol. The signals of β-catenin and other cytosolic proteins, including ERK1/2 and AKT, on the blots were quantified (right). *n* = 3; two-tailed paired multiple t-test. T; total, C; cytosol, N; nucleus, M; mitochondria. n.s.; non-significant. **B–D** Co-immunostaining of β-catenin and TOM20 (**B**) or AK3-HA (**C**) in A549 cells. Images were obtained by super resolution using Airyscan 2 (**B**) or confocal microscopy (**C**), and stacked into z-position (left below; z-stack position). Scale bar; 10 μM (**B**) or 5 μM (**C**). Co-localization between β-catenin and Mitotracker or AK3-HA was analyzed and presented as the percentage of co-localization in HCT116 cells (D). Two-tailed unpaired t-test. (**E**,**F**) Nuclear β-catenin levels following Wnt activation and AK3 overexpression. HCT116 cells were transfected with AK3-HA, incubated with 100 ng/ml recombinant Wnt3a for 16 h, and then treated with 10 μM CCCP for additional 4 h. Images of the immunostained cells were obtained by confocal microscopy (**E**), and Pearson’s coefficients between β-catenin and Hoechst signals were represented as the percentage of co-localization (**F**). **G** AK3 is an enzyme recycling guanosine nucleotide demanded for TCA cycle. (**H**,**I**) Generation of AK3-∆1 and AK3-∆2 mutants losing their abilities to bind AMP and GTP, respectively (**H**). TOPflash/FOPflash assay to analyze their effects on β-catenin signaling in A549 cells (**I**). AK4 was utilized as a control. *n* = 5. ns; non-significant. **J** MMP (ΔΨm) alteration in A549 cells expressing AK3 WT and mutants. Cells were left untreated (NT) or treated with 10 μM CCCP for 2 h, or transfected with AK3 for 24 h. Cells were stained with Mitotracker Red CMXRos and analyzed by flow cytometry. *n* = 4. (**K**) Proportion of mitochondrial ATP in A549 cells expressing AK3 WT and mutants. Cells were transfected with AK3 for 24 h and the ATP levels were measured. Ratio of ATP^OxPhos^ to ATP^Total^ was calculated and statistically analyzed. *n* = 3. **L** Proportion of mitochondrial ATP in A549 AK3-KO cells. ATP levels were measured after 48 h. Ratio of ATP^OxPhos^ to ATP^Total^ was calculated and statistically analyzed. (*n* = 3, two-tailed paired t-test). Bars represent mean ± s.d. (**A**) or mean ± s.e.m. (**D**,**F**,**I–L**). One-way ANOVA followed by dunnett’s multiple comparison tests (**F**,**I–K**). (*P < 0.05, **P < 0.01, ***P < 0.001, ****P < 0.0001).

Since both AK3 level and OxPhos pathway show a negative correlation with β-catenin signaling, we reasoned that AK3 expression regulates β-catenin signaling via the mitochondrial event. We first analyzed the distribution of β-catenin with fractionation assays. Notably, we found abundant β-catenin in the mitochondria-enriched fraction as well as in the nucleus-enriched fraction of A549 cells (Fig. 3A). Ectopic expression of AK3 significantly increased β-catenin levels in the mitochondrial fraction, while it reduced it in the nuclear fraction (Fig. 3A and Fig. S3A). Subcellular distribution of other cytosolic proteins, including ERK1/2 and AKT, was not affected by AK3 expression. The β-catenin levels decreased in the mitochondrial fraction but increased in the nuclear fraction of A549 AK3-KO cells, which possibly enhanced the LEF1-β-catenin interaction in these cells (Figs. S2E and S3B). Using super‑resolution microscopy, we could observe partial co‑localization of β‑catenin with the mitochondrial marker TOM20 in A549 cells (Fig. 3B). This co‑localization was more pronounced in the central region of cell body than at the basal surface. Furthermore, overexpression of AK3, but not AK4, enhanced co-localization of β-catenin with mitochondria (Fig. 3C, D and Fig. S3C). Recombinant Wnt3a treatment induced nuclear β‑catenin accumulation in HCT116 cells (which harbor both CTNNB1^WT^ and CTNNB1^S45del^), but this accumulation was significantly reduced in cells overexpressing AK3 (Fig. 3E, F). These results suggest that nuclear translocation of β-catenin may be regulated by the mitochondria expressing AK3.

To determine whether AK3 enzymatic activity is required for its regulation of β‑catenin signaling, we generated two AK3 mutants: an AMP‑binding–null mutant (∆1) and a GTP‑binding–null mutant (∆2) (Fig. 3G, H and figure S3D) [29]. Unlike AK3 WT, neither AK3 ∆1 nor AK3 ∆2 was able to suppress β‑catenin signaling in A549 cells (Fig. 3I). In HeLa cells, AK3 ∆2 showed significantly decreased co-localization of β-catenin with mitochondria compared with AK3 WT, but higher co-localization than that in non-transfected cells, possibly reflecting its partial activity in HeLa cells, but not in A549 cells (figure S3C-F). In these assays, enzymatically inactive mitochondrial AK4 was used as a negative control. Next, to confirm that AK3 catalytic function enhances mitochondrial activity, we measured mitochondrial membrane potential (MMP) which reflects mitochondrial functionality [39]. Compared to control cells, expression of AK3 WT, but not AK3 ∆1 or ∆2 mutant, significantly increased MMP in A549 cells (Fig. 3J). Treatment with the uncoupler carbonyl cyanide m‑chlorophenyl hydrazone (CCCP), which dissipates MMP [40] (Fig. 3J), abolished the inhibitory effects of AK3 on the nuclear β-catenin accumulation (Fig. 3E, F). A549 AK3-KO cells also exhibited lower MMP than control cells (Fig. S3G). Further, measurement of mitochondrial ATP production (in the presence of the glycolysis inhibitor 2‑deoxy‑D‑glucose) showed that AK3 WT, but not AK3 ∆1 or AK3 ∆2, enhanced mitochondrial ATP output (Fig. 3K), whereas AK3 KO cells displayed reduced mitochondrial energy dependency (Fig. 3L). These results demonstrate that the catalytic activity of AK3 suppresses nuclear β‑catenin accumulation and its signaling.

### Mitofusins mediate the inhibitory effect of AK3 on β-catenin signaling

**Fig. 4:**
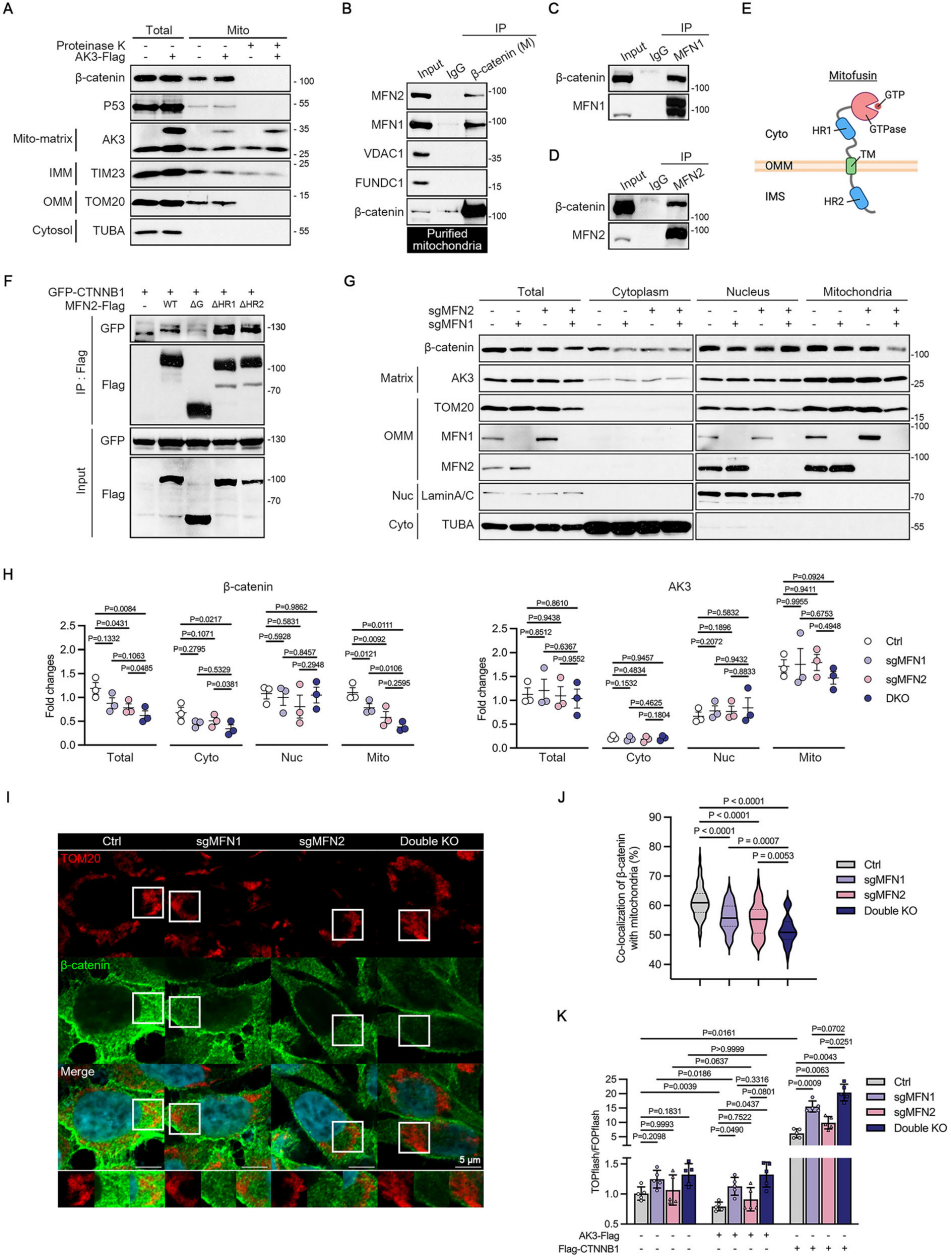
AK3 suppresses β-catenin signaling through a mitofusins-dependent mechanism. **A** Proteinase K assay in A549 cells. After transfection of A549 cells with AK3-Flag for 24 h, mitochondria were purified, incubated with 100 μg/ml proteinase K for 30 min, and analyzed by western blotting. Mito-matrix; mitochondrial matrix, IMM; inner mitochondrial membrane, OMM; outer mitochondrial membrane. **B–D** Immunoprecipitation (IP) assay to identify the β-catenin-interacting proteins. Purified mitochondria from HEK293T cells were subjected to IP assay using anti-β-catenin (**B**), anti-MFN1 (**C**), and anti-MFN2 (**D**) antibodies. IgG was used as a negative control. Input (5%). **E**,**F** Mapping the mitofusin domain that interacts with β-catenin. Schematic diagram showing mitofusin domains, GTPase (**G**) domain, first heptad repeat (HR1) domain, transmembrane (TM) domain, and second heptad repeat (HR2) domain (**E**). HCT116 cells were transfected with GFP-CTNNB1 WT and MFN2-Flag WT or mutant for 24 h and subjected to IP assay using Flag antibody (**F**). **G**,**H** Subcellular fractionation assay in HCT116 MFN1-KO, MFN2-KO, and double KO cells. Markers for the indicated subcellular organelles are shown (**G**, left). Signals on the blots were quantified and statistically analyzed (**H**). Bars represent mean ± s.e.m. *n* = 3. **I**,**J** Co-localization of β-catenin and TOM20 in HCT116 MFN1-KO, MFN2-KO, and double KO cells. Cells were immunostained with anti-β-catenin and anti-TOM20 antibodies and observed by confocal microscopy. Scale bars; 5 μm (**I**). Pearson’s coefficients between the fluorescent signals were presented as the percentage of co-localization. Bars represent mean ± s.e.m. *n* = 67, 47, 58, 36 (**J**). **K** TOPflash/FOPflash assay to analyze the effects of AK3 expression on β-catenin signaling in HCT116 MFN-KO cells. HCT116 WT and MFN-KO cells were transfected with AK3-Flag or Flag-CTNNB1 for 24 h. Bars represent mean ± s.e.m. *n* = 5. Two-way ANOVA followed by multiple comparison tests using Tukey’ correction. Paired one-way ANOVA followed by dunnett’s multiple comparison tests (**H**,**J**). (*P < 0.05 **P < 0.01, ***P < 0.001, ****P < 0.0001).

The proteins containing Armadillo (ARM) repeat, such as ARMC1, SARM1, and ARMC10, can interact with outer membrane of mitochondria (OMM) proteins in response to various stimuli [41–43]. We therefore hypothesized that β‑catenin, which contains 12 ARM repeats, also interacts with OMM proteins. Using proteinase K assay, we confirmed that β‑catenin does not localize inside mitochondria (Fig. 4A). To identify the mitochondrial interactors of β-catenin, we performed an immunoprecipitation (IP) assay on the mitochondria purified from HEK293T cells expressing Flag‑β‑catenin. Both mitofusin 1 and 2 (MFN1 and 2), but not other OMM proteins, such as VDAC1 and PGAM5, interacted with Flag-β‑catenin. (Fig. S4A). Reciprocal IP assays following transfection with MFN1‑Flag and MFN2‑Flag confirmed these interactions (Fig. S4B). We validated the interaction between endogenous β-catenin and MFN1 or MFN2 in HEK293T cells (Fig. 4B–D). The β‑catenin-MFN interactions were also observed in A549 cells (Fig. S4C). In addition, to map the β‑catenin–binding region of MFN2, we tested MFN2 deletion mutants. Both MFN2 ∆HR1 and ∆HR2 (lacking the heptad repeat domains) bound to β‑catenin at levels comparable to MFN2 WT, whereas MFN2‑∆G mutant (lacking the GTPase domain) showed minimal interaction (Fig. 4E, F). The GTPase domain was also crucial for the β-catenin-MFN1 interaction (Fig. S4D). These data indicate that the GTPase domain of MFNs is required for binding to β‑catenin.

Next, we generated HCT116 MFN1‑KO, MFN2‑KO, and MFN1/2 double‑KO cells using CRISPR/Cas9 system (Fig. S4E–G). As reported [44], MFNs-deficiency led to shorten and fragmented mitochondrial morphologies which imply the loss of mitochondrial integrity (Fig. S4H). In both HCT116 (Fig. 4G, H) and HeLa cells (figure S4I), β‑catenin levels in mitochondrial fraction were markedly reduced by MFNs knockout, and co‑localization of β‑catenin with TOM20 was diminished in HCT116 MFNs-KO cells (Fig. 4I, J). Meanwhile, total β-catenin levels decreased by MFNs KO in HCT116 and HeLa cells (Fig. 4G, H and Fig. S4I). In addition, AK3 overexpression in the presence of cycloheximide enhanced protein stability of β-catenin (Fig. S4J). Together with the observed increase in β-catenin protein levels in AK3-overexpressing cells (Fig. 2B), we suggest that mitochondria regulate not only β-catenin signaling but also β-catenin protein stability.

We then evaluated the AK3-inhibitory effect on β-catenin signaling in MFNs-KO cells. While AK3 overexpression consistently inhibited β-catenin signaling in HCT116 WT cells, this inhibition was attenuated in HCT116 MFN1-KO and MFN2-KO cells, and completely abolished in HCT116 double-KO cells (Fig. 4K). According to the report [45], MFN2 knockdown (KD) in human lung microvascular endothelial cells markedly amplified β-catenin signaling in response to TNFα treatment, as demonstrated by the TOPflash assay. Similarly, we could observe that β-catenin signaling was significantly enhanced in all types of MFN-KO cells upon Flag-CTNNB1 overexpression (Fig. 4K). Analysis of LUAD patient data (GSE31210) revealed that MFN1‑ and MFN2‑associated gene signatures are significantly enriched for β‑catenin signaling-related GO terms (figure S4K, L). Together, these data imply that mitofusins are required for the AK3-mediated inhibition of β-catenin signaling activation and also mediate the mitochondria-mediated protein stability regulation of β-catenin.

### Mitochondrial network morphology is influenced by β-catenin

**Fig. 5:**
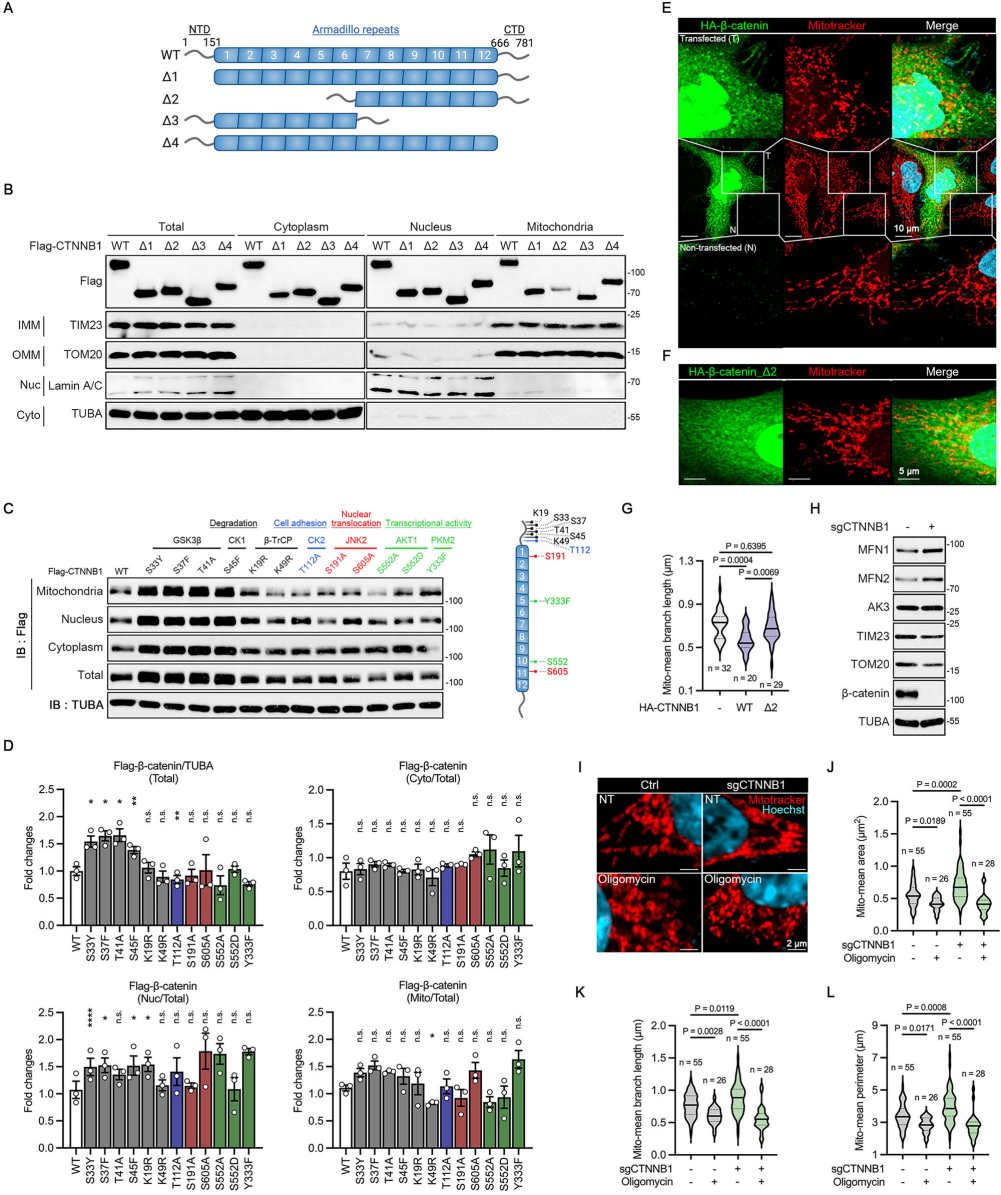
β-catenin influences mitochondrial dynamics through ARM repeats 1 to 6. **A** Generation of β-catenin deletion (∆) mutants. **B–D** Subcellular fractionation assays to analyze localization of β-catenin deletion mutants (**B**) and PTM-defective mutants (**C**). HEK293T cells were transfected with β-catenin mutants for 24 h. Marker proteins of subcellular organelles are indicated. Schematic diagram presents the modification residues of β-catenin (**C**, right). Signals on the blots were quantified and statistically analyzed (**D**). Bars represent mean ± s.e.m. *n* = 3. **E–G** Immunostaining of β-catenin WT or ∆2 mutant with mitotracker staining in HeLa cells. HeLa cells transfected with β-catenin WT (**E**) or ∆2 mutant (**F**) were stained with anti-β-catenin antibody and mitotracker. Mitochondrial branch length (**G**) was analyzed by confocal microscopy and quantified by Image J. Scale bars; 10 μm (**E**) and 5 μm (**F**). Violin plots show frequency distribution and median. **H–L** Generation of HCT116 CTNNB1-KO cells. Immunoblot analysis was performed on HCT116 cells (**H**), and mitochondrial morphologies of these cells were observed under confocal microscope after staining with mitotracker (**I**). Cells were treated with 1 μg/ml oligomycin A for 2 h to induce mitochondrial fragmentation. Scale bars; 2 μm. Mitochondrial area (**J**), branch length (**K**), and perimeter (**L**) on the confocal images were quantified by Image J. Violin plots show frequency distribution and median. One-way ANOVA followed by dunnett’s multiple comparison tests (**D**,**G**,**J–L**). (*P < 0.05 **P < 0.01, ***P < 0.001, ****P < 0.0001).

As scaffold domains, ARM repeats in β-catenin mediate various protein-protein interactions, including interactions with cadherins, APC, and TCF/LEF [36]. To investigate the role of ARM repeats in β-catenin localization, we generated four types of β-catenin mutants, each lacking one of the following domains; the N-terminal domain (NTD) (∆1), ARM repeats 1-6 (∆2), ARM repeats 7-12 (∆3), and the C-terminal domain (CTD) (∆4) (Fig. 5A). Of note, unlike other mutants, β-catenin ∆2 mutant barely localized in the mitochondrial fraction (Fig. 5B). Flag-tagging at the CTD or NTD did not alter subcellular localization of β-catenin (Fig. S5A). In addition, β-catenin ∆2 mutant could not bind to both the MFN1 and MFN2 (Fig. S5B).

To examine subcellular localization of β-catenin point mutants on the residues for post-translational modification, which mediate various signaling and regulate the β-catenin protein stability [36], we performed fractionation assay and normalized their amounts in a subcellular fraction relative to total β-catenin. The results revealed no significant changes in their mitochondria localization except K49R mutant which significantly decreased in the mitochondria fraction. Since K49 residue of β-catenin is a ubiquitination residue, mitochondria may preferably bind and affect stability of the ubiquitinated β-catenin protein [36]. The β-catenin S33Y, S37F, S45F, and K19R mutants seemed to better translocate into the nuclear fraction without significant change in the cytoplasmic fraction (Fig. 5C, D and Fig. S5C, D). Together, mitochondria interact with most of the unmodified β-catenin as well as some modified β-catenin (Fig. 2A, B). These results also highlight the importance of β-catenin ARM repeats 1-6 (a.a. 151-389) as scaffold domains for the β-catenin–MFN interaction.

The notion that mitofusins are involved in mitochondrial fusion led us to assess whether β-catenin-mitofusins interactions influence mitochondrial dynamics. Interestingly, in HeLa cells which are widely used for mitochondrial dynamics studies [46], overexpressing β-catenin showed fragmented and short morphologies of mitochondria compared to control cells (Fig. 5E, G). On the other hand, HeLa cells expressing β-catenin ∆2 mutant did not exhibit the fragmented mitochondria pattern, with no significant differences from control cells (Fig. 5F, G). We also generated HCT116 CTNNB1-KO cells and analyzed their mitochondrial morphologies. In HCT116 CTNNB1-KO cells, MFN1 and MFN2 protein levels were upregulated even though levels of other mitochondrial proteins, including TOM20 and TIM23, decreased (Fig. 5H). Estimation of mitochondrial morphologies under confocal microscope revealed that mitochondrial network was more elongated in CTNNB1-KO cells than control cells (Fig. 5I–L). In this assay, oligomycin A was used as a control to induce and confirm mitochondrial fragmentation in all cells. Overall, β-catenin might interfere with mitochondrial fusion through ARM repeats.

### The β-catenin-mitofusins interactions are affected by AK3 expression

**Fig. 6:**
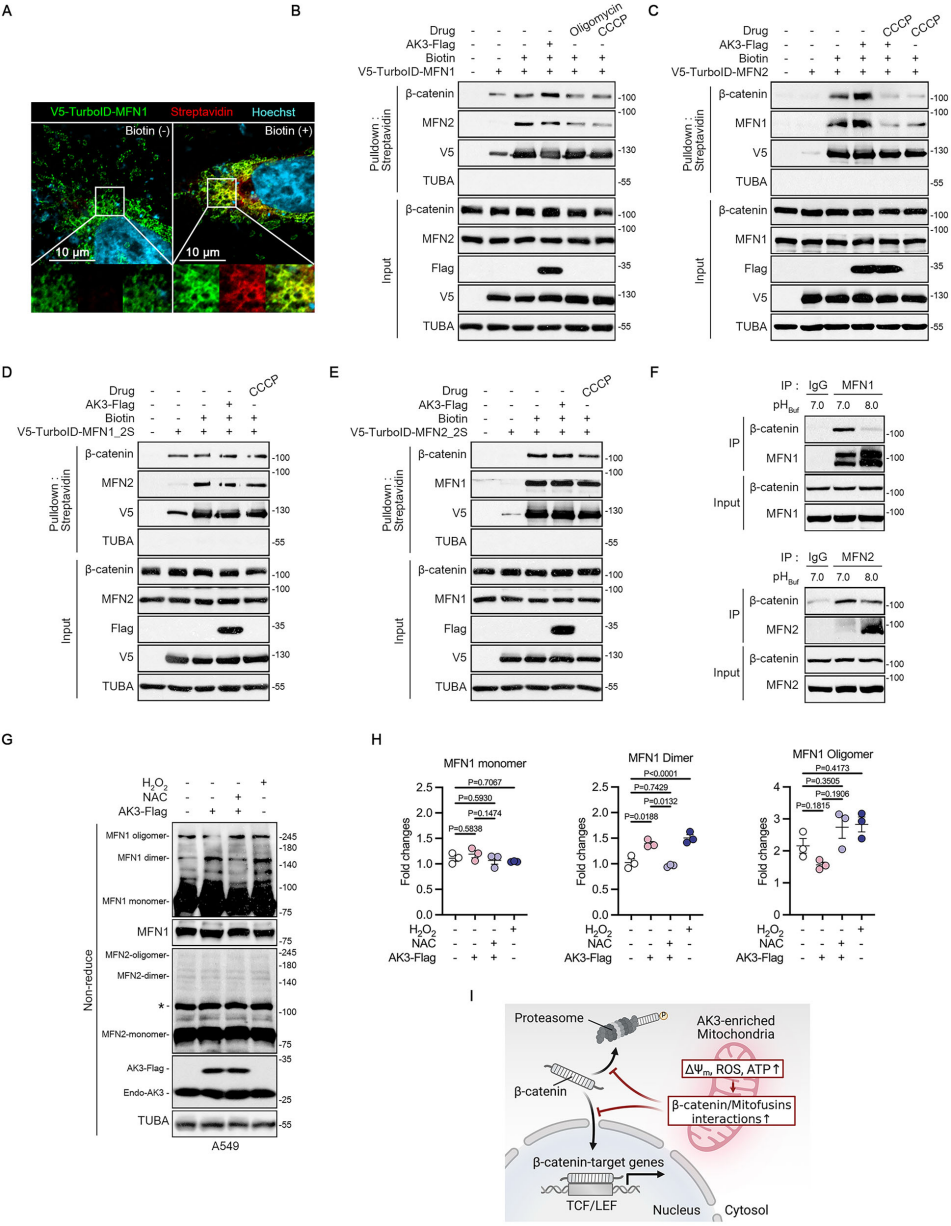
Mitochondrial activity enhancement induced by AK3 modulates β-catenin-mitofusins interactions. **A** Visualization of biotinylated proteins by streptavidin-staining. HeLa cells expressing V5-TurboID-MFN1 were incubated with 50 μM biotin for 1 h and stained with anti-V5-tag antibody and streptavidin. Scale bars; 10 μM. **B**,**C** Streptavidin-pulldown assay in HeLa cells expressing V5-TurboID-MFN1 (**B**) or V5-TurboID-MFN2 (**C**). Cells were transfected with AK3-Flag for 24 h or treated with 1 μg/ml oligomycin A or 10 μM CCCP for 1 h. After incubated with 50 μM biotin for 1 h, cell lysates were pulled-down with streptavidin. Biotinylated proteins were analyzed by immunoblotting. **D**, **E** Streptavidin-pulldown assay for assessing the interaction between β-catenin and V5-TurboID-MFN1-2S (**D**) or V5-TurboID-MFN2-2S (**E**). HeLa cells were transfected with V5-TurboID-MFN1-2S or V5-TurboID-MFN2-2S alone, or together with AK3-Flag for 24 h, exposed to 10 μM CCCP for 1 h, and then subjected to pulled-down assay using streptavidin. **F** IP assays were performed in HCT116 cells using IP buffers adjusted to pH 7.0 or pH 8.0 to assess interactions between β‑catenin and MFN1 and MFN2. **G**,**H** MFN1 monomer, dimer, and oligomer formation in A549 cells. A549 cells were transfected with AK3-Flag for 24 h with or without pre-treatment with 20 mM NAC or 500 μM H_2_O_2_ for 30 min. Cells were lysed and separated by PAGE under non-reducing condition and analyzed by western blot. Asterisk indicates non-specific signal (**G**). Signals of MFN1 monomer, dimer, and oligomers on the blots were quantified, normalized to TUBA, and statistically analyzed (**H**). One-way ANOVA followed by dunnett’s multiple comparison tests. Bars represent mean ± s.e.m. (*n* = 3). **I** Hypothetical model showing a role of AK3 in the β-catenin signaling. AK3-enriched mitochondria exhibit increase in MMP, ROS generation, and ATP production, which in turn enhances the β-catenin–mitofusins interactions. These effects collectively suppress both nuclear signaling and proteasomal degradation of β-catenin.

To further investigate whether the interactions between β-catenin and mitofusins are influenced by AK3, we generated TurboID-MFN fusion proteins for a TurboID assay, which allows detection of a temporal binding state of proximal proteins through the biotinylation [47]. V5-TurboID-MFN1 and -MFN2 proteins were all found in the mitochondria (Fig. S6A). Immunoblotting of the biotinylated proteins pulled-down by streptavidin confirmed the expression and functionality of V5-TurboID-MFNs (Fig. S6B, C). Immunostaining assay also exhibited overlapping signals between V5 and streptavidin after the supplementation with biotin (Fig. 6A and Fig. S6D). As mitofusins are known to form homo and hetero-dimers [48], we also found that V5-TurboID-MFNs biotinylated V5-TurboID-MFNs itself and its partner MFNs in HeLa cells (Fig. 6B, C). Consistent to the results of IP assays, β-catenin was biotinylated as an V5-TurboID-MFNs-interacting protein and the biotinylated β-catenin increased upon AK3 overexpression. Of note, disrupting mitochondrial function by treatment with oligomycin A or CCCP reduced the biotinylation of β-catenin as well as MFN1 and MFN2 in TurboID assay (Fig. 6B, C).

Given that no direct interaction was observed between AK3 and β-catenin (Fig. S4A), we proposed that the mechanism underlying the role of AK3 in regulating the MFN-β-catenin interaction involves MFN sulfenylation–associated dimerization of MFN. It was previously shown that elevated ROS levels in the mitochondrial intermembrane space (IMS) promote sulfenylation of two cysteine residues in MFN2 C-terminus, enhancing MFN2 dimerization to drive mitochondrial fusion [49]. Similarly, TNFα-induced ROS triggers MFN2 sulfenylation (MFN2–SOH) to promote the MFN2 dimerization and the MFN2–β-catenin interaction [45]. Consistent with these reports, substitution of the two cysteine residues (MFN1; C665, 681 and MFN2; C684, C700), which are essential for MFN1 and MFN2 dimerization [49], with serine (MFN1-2S and MFN2-2S) abolished the AK3–induced increase in the MFN–β-catenin interaction (Fig. 6D, E and Fig. S6E), suggesting that MFN sulfenylation is essential for AK3 to modulate the MFN–β-catenin interaction. In addition, we observed that AK3 overexpression increased superoxide levels as well as mitochondrial ATP production and MMP (Fig. 3J, K, Fig. S6G). Mitochondrial dysfunction induced by AK3-KO or treatment with rotenone or CCCP also elevated mitochondrial ROS, but decreased MMP and OxPhos-ATP production and blocked the MFN-β–catenin interaction (Figs. 3L, 6B, C and Fig. S3G, S6F). These results suggest that other factors as well as ROS elevation could also regulate the MFN–β-catenin interaction.

The higher MMP leads to more acidic pH_IMS_ and this acidic condition affects the protein charges, conformational state, and proteolytic processing in IMS [50–54]. We thus hypothesized that pH and ROS in the IMS coordinate the AK3-induced regulation of MFN–β-catenin interaction. Supporting this, we observed that the MFN1/2–β-catenin interactions markedly increased at pH 7.0 compared to pH 8.0 conditions (Fig. 6F). Furthermore, with western blotting following PAGE under non-reducing conditions, we found that MFN1 dimer formation significantly increased by AK3 overexpression or hydrogen peroxide treatment, whereas restored by antioxidant NAC treatment to the control levels (Fig. 6G, H), indicating that AK3-induced redox environment promotes MFN1 dimerization. Under this condition, there were no significant changes in MFN1 monomer and oligomer levels, and it was difficult to detect MFN2 dimers and oligomers. Using DCP-bio1 probe, we also analyzed protein sulfenylation and observed that α-tubulin and MFN1 sulfenylation levels were higher at pH 7.0 than pH 8.0 condition, suggesting that pH affects MFN1 sulfenylation (Fig. S6H). These results suggest that AK3-mediated regulation of the MFN–β-catenin interaction is coordinated by redox state and pH within the IMS, which are coupled with outcomes of the increased mitochondrial activity (Fig. 6I and Fig. S6I).

In cancer cells, β-catenin is highly up-regulated for tumorigenesis. We thus asked whether supplementation with mitochondria reduces β-catenin signaling in cancer cells. Mitochondria were purified from human embryonic kidney HEK293T cells and then transplanted into HCT116 cancer cells. Fine staining with Mitotracker Red showed that the transplanted mitochondria maintained MMP and remained functional in HCT116 cells (Fig. S7A). Interestingly, the recipient HCT116 cells showed reduction in cell proliferation and TCF/LEF activity compared to control cells (Fig. S7B, C). With western blot assay, we found that TCF1/7 and Cyclin D1 levels significantly decreased in the recipient HCT116 cells compared to control cells (Fig. S7D). The exogenous TOM20 originated from HEK293T cells was detected in HCT116 cells after the transplantation. Mitochondrial proteins AK3 and TOM20, and β-catenin levels all significantly increased in the recipient HCT116 cells (figure S7D). These observations reinforce our proposal that functional mitochondria inhibit β-catenin signaling and provide new therapeutic opportunities to supplement mitochondria.

## Discussion

Our study provides the evidence how the mitochondria interact with β-catenin signaling pathway. Mitochondrial enzyme AK3, which is involved in the TCA cycle and OxPhos, shows a highly inverse correlation with β-catenin signaling. This role of AK3 depends on its enzyme activity which recycles GTP for maintaining the TCA cycle, demonstrating that mitochondrial metabolism, including OxPhos pathway, is important for the activation of β-catenin. Similarly, the tumor-suppressive role of mitochondrial pyruvate carrier (MPC) in Wnt/β-catenin signaling underlies the importance of pyruvate metabolism for TCA cycle in determining cell fate and proliferation [14, 15, 55]. While more direct evidence remains to be clarified, mitofusins can provide the link connecting the mitochondrial metabolism to β-catenin signaling through the β-catenin-mitofusins interactions (Fig. 6I), suggesting a tumor suppressive role of AK3.

Given the G domain of mitofusins is crucial for MFN dimerization in the mitochondrial fusion process, the binding of β-catenin to the G domain is expected to interfere with GTPase activity and thereby mitochondrial fusion. The β-catenin–MFN2 interaction was also reported to occur at the contact site between mitochondria and adherens junction [45]. This interaction is enhanced in endothelial cells upon inflammatory stimuli through the MFN2 sulfenylation followed by its dimerization, which is induced by high ROS levels, and suppresses β-catenin signaling in a non-canonical manner. Our results similarly showed that the two sulfenylation sites and the MFN dimerization are important for the β-catenin–mitofusins interactions related to mitochondrial activity. Since the protein disulfide bond formation in the IMS is highly pH_IMS_-dependent [56, 57], the redox state, pH, and ATP levels within the IMS might collectively determine MFN protein structures (Fig. S6I). It has been largely unknown how the MFNs sense the metabolically active state of mitochondria and signal their states to the nucleus in transcriptional level. Thus, we believe that the AK3–MFN–β-catenin axis can provide a new insight and better understanding of mitochondria biology.

The role of mitofusins appears analogous to that of E-cadherin in inhibiting β-catenin signaling. E-cadherin^−/−^ embryonic stem cells show decrease in total β-catenin levels but increase in the β-catenin–LEF1 interaction, mimicking Wnt activation. It was proposed that free β-catenin released from E-cadherin undergoes either proteasomal degradation or nuclear signaling activation [9, 58–60]. Similar to this non-canonical pathway, our results reveal that cells lacking both mitofusins could not inhibit the β-catenin signaling upon AK3 overexpression. Simultaneously, total β-catenin levels decrease following genetic ablation of mitofusins, suggesting an additional facet of the β-catenin–mitofusins interactions interfering with proteasomal degradation of β-catenin. Intriguingly, we found that E-cadherin levels were reduced in AK3-KO cells, which might also lead to β-catenin release from the β-catenin–E-cadherin complexes to increase β-catenin signaling. Nonetheless, we have confirmed that β-catenin interacted with mitofusins in various ways and AK3 expression truly modulated the β-catenin–mitofusins interactions. Therefore, we propose that AK3 manipulates mitochondrial localization of β-catenin to directly regulate the signaling, as E-cadherin does. The fate of β-catenin where to be localized among nucleus, mitochondria, and adherens junction remains unclear and requires further investigation. This non-canonical pathway to regulate both β-catenin signaling and protein stability oppositely would be an intriguing topic.

*CTNNB1* is one of the most commonly mutated genes among the cancers and 80% of colorectal cancer patients harbor mutations in the *APC* gene [31, 32]. Wnt/β-catenin signaling is highly activated in 50% of human non-small cell lung cancers (NSCLC) [30]. While clinical trials of inhibitors targeting the Wnt pathway have been conducted, no applicable drugs have been developed yet [61, 62]. Given whole genome and RNA sequencings are increasingly adopted for the prognosis and diagnosis of cancer patients, our results provide an advanced insight which counts mitochondrial metabolic states, including *AK3* expression and OxPhos score, for the individualization of cancer therapy, especially in Wnt-activated patients. Adopting the metabolites and small molecules to enhance mitochondrial activity can be an option for curing these patients. Unlike traditional chemotherapies and target therapies, promoting mitochondrial respiration is expected to present less side effects. Alternatively, mitochondrial transplantation with enhanced cell fitness can also offer a potential opportunity for a novel approach [63, 64]. Recent studies reported that mitochondrial respiration is restored in metastatic tumor cells to adapt the TME of metastatic site [3, 34]. Thus, focusing on metabolic differences between primary and metastatic tumors, or solid and liquid tumors, will be valuable for a better understanding of cancer.

In addition, comprehensive understanding the role of mitochondria in Wnt/β-catenin signaling will also be important for understanding tissue repair, cardiovascular diseases, neurodevelopmental disorders, and neurodegenerative diseases [65–68]. In particular, stemness of embryonic and adult stem cells is primarily dictated by the spatiotemporal regulation of Wnt/β-catenin signaling [69]. We expect that modulating mitochondrial metabolism not only impedes cancer progression, but also revives stemness and prevents defects in differentiation.

## Methods

### Microarray and RNA sequencing datasets analysis

Normalized gene expressions were obtained from The Cancer Genome Atlas (TCGA) database (http://firebrowse.org) and Gene Expression Omnibus (NCBI-GEO, https://www.ncbi.nlm.nih.gov/geo/), transformed into log2 scale, and compared between tumor and matched non-tumor tissues. Kaplan–Meier survival curves were determined by calculating significant *P* values between the groups. For each marker (AK3, OxPhos, Wnt/β-catenin scores), patients were divided into low/high groups at the cutoff that minimized the log-rank *P* value. The *P* value for every cutoff was calculated using R. *AK3* gene signature was determined by assessing Pearson’s coefficients between *AK3* expression (224151_s_at) and the other genes. The *AK3* associated genes were ranked by their Pearson’s coefficients and the significantly correlated genes (R ≤ -0.3 or R ≥ 0.3) were referred as *AK3* gene signature. Gene ontology analysis was performed on the *AK3* gene signature using ‘DAVID’ bioinformatic tool (https://david.ncifcrf.gov) [70]. Gene set enrichment analysis (GSEA) software (RRID:SCR_003199) and MSigDB (https://www.gsea-msigdb.org/gsea) were used to identify the molecular signatures of the *AK3* gene signature.

### OxPhos and signaling pathway scores calculation

Pathway scores were calculated as previously described [34]. The following list of pathway genes was created based on this; OxPhos genes from the KEGG_OXIDATIVE_PHOSPHORYLATION gene set, and the genes of the PDH complex (PDHA1, PDHB, DLAT, DLD, and PDHX); Wnt/β-catenin genes from the KEGG_WNT_SIGNALING_PATHWAY gene set; PI3K/AKT/mTOR genes from the HALLMARK_PI3K_AKT_MTOR_SIGNALING gene set; and P53 genes from the HALLMARK_P53_PATHWAY gene set. Gene expression of the gene sets was log2-transformed, mean-centered, and z-transformed. Principal component analysis (PCA) was performed, and the first principal component (PC1) was used as the pathway score.

### Cell culture and transfection

HEK293T (RRID: CVCL_0063), A549 (RRID: CVCL_0023), HCT116 (RRID: CVCL_0291), and HeLa cells (RRID: CVCL_0030) provided by ATCC showed negative results on mycoplasma contamination tests. Cells were cultured in DMEM (Welgene, LM001-05) or RPMI1640 (Cytiva, SH30027.01) supplemented with 10% FBS (Gibco, 16000044), 0.5% penicillin/streptomycin (Gibco, 15140122), and 0.05% gentamicin (Gibco, 15750060). Transfection was performed with Lipofector-pMAX (AptaBio, AB-LF-M100) following the manufacturer’s instructions.

### Generation of stable KO cell lines

LentiCRISPR v2 plasmid (RRID: Addgene_52961) was reconstructed to target the gene of interest using the CRISPR/Cas9 system. Single guide RNAs (sgRNAs) specific to the target gene were designed by CRISPick (https://portals.broadinstitute.org/gppx/crispick/public) and validated target sequences by Feng Zhang lab [71]. sgAK3 #1: 5′-AAC GCC TTA CTG CTC GC-3′, sgAK3 #2: 5′-GTT ATA GAC TCG GCC ACT GG-3′, sgMFN1 #1: 5′-CAC CAG GTC ATC TCT CAA GA-3′, sgMFN1 #2: 5′-TAA GCA CAT AGA GGA TGG TA-3′, sgMFN2 #1: 5′-GGT TAC CTA TCC AAA GTG AG-3′, sgMFN2 #2: 5′-ACT TAA GCA CTT TGT CAC TG-3′, sgCTNNB1: 5′-AAG GTT ATG CAA GGT CCC AG-3′. Cells were transfected with each plasmid, selected with 1 μg/ml puromycin for 48 h, and the knockout cells were further isolated at single-cell level. For the generation of HCT116 DKO cells, HCT116 MFN2-KO cells were additionally transfected with sgRNA targeting MFN1.

### Plasmid constructs

AK3 (#37539), MFN1 (#141154), and MFN2 (#139192) cDNA plasmids were obtained from Addgene. CTNNB1 cDNA, TOPflash, and FOPflash plasmids were gifts from S.H. Baek (Seoul National Univ. Korea). The pcDNA3.1-HA (Addgene Plasmid #128034, RRID: Addgene_128034), p3xFLAG-CMV-10 (Sigma Aldrich, E7658), p3xFLAG-CMV-14 (Sigma Aldrich, E7908), pEGFP-C1 (NovoPro, V012024), and pEGFP-N1 (NovoPro, V012021) were modified to fuse tags (HA, FLAG, or GFP) to either the N-terminus or C-terminus of AK3, MFN1, MFN2, or β-catenin. V5-TurboID-pcDNA5 plasmid was a gift from H.W. Rhee (Seoul National Univ. Seoul, Korea). AK3, MFN1, MFN2, and CTNNB1 mutants were generated by site-directed deletion and single site mutagenesis and verified by DNA sequencing.

### Immunoblotting

Western blot assay was performed as previously described [26]. Antibodies against AK2 (Santa Cruz Biotechnology (SCBT), sc-28786, RRID:AB_2225292), AK3 (Proteintech, 12562-1-AP, RRID:AB_2305373), AK4 (SCBT, sc-271161), α-tubulin (SCBT, sc-23948), CDC42 (BD Transduction Laboratories, 610929), RAC1 (BD Transduction Laboratories, 610650), GSK-3β (ABclonal, A11731-20), p-GSK-3β (S9) (Cell Signaling Technology (CST), 5558), β-catenin (SCBT, sc-7963), AKT (CST, 9272), P44/42 MAPK (CST, 9101), HSP60 (SCBT, sc-1052), TIM23 (BD Transduction Laboratories, 611222), Lamin A/C (SCBT, sc-376248), FLAG M2 (Sigma-Aldrich, F1804), P53 (SCBT, sc-126), TOM20 (SCBT, sc-17764), MFN1 (ABclonal, A21293), MFN2 (ABclonal, A19678), VDAC (SCBT, sc-390996), FUNDC1 (Aviva systems biology, ARP53280_P050), GFP (SCBT, sc-8334), streptavidin-HRP (Sigma, RABHRP3), V5 (Abcam, ab27671), and PGAM5 (ABclonal, A22203) were used for the assay.

### Cell proliferation and migration

To assess cell proliferation using the MTT assay (ThermoFisher Scientific, M6494), cells were seeded into a 96-well plate and analyzed after 48 h. The cells were incubated with 0.5 mg/ml MTT in serum-free media for 3 h at 37 °C. MTT solvent (4 mM HCl, 0.1% NP40 in isopropanol) was added, and the plate was further incubated for 15 min at RT. Absorbance was measured at 590 nm using a Multiskan FC (ThermoFisher Scientific). To analyze cell proliferation by cell counting, 1×10⁴ A549 cells or 6×10⁴ HCT116 cells were seeded in a 6-well plate, and cell numbers were counted on the indicated days. A wound healing assay was performed to assess the migratory ability of cancer cells. Cells were seeded into a 6-well plate to achieve 100% confluency, and a scratch was made using a 200 μl pipette tip. The scratched area was imaged after 24–28 h and quantified using ImageJ.

### β-catenin transcriptional activity

A TOPflash assay was performed to analyze β-catenin transcriptional activity [37]. Cells were transfected with a combination of TOPflash or FOPflash, along with p3xFLAG-CMV-14, AK3-Flag, Flag-CTNNB1, or AK4-Flag plasmids. After 24 h, cells were detached using trypsin-EDTA, lysed, and analyzed using the Luciferase Assay System (Promega, E1500) according to the manufacturer’s instructions. Recombinant human Wnt3a protein (Proteintech, HZ-1296) was added 4 h after transfection and incubated for 16 h. Luminescent signals were measured with a Lumat LB9507 luminometer (Berthold). The ratio of TOPflash to FOPflash was calculated and presented as β-catenin transcriptional activity.

### RNA isolation and quantitative real time PCR

RNA was isolated using TRI reagent (Genbiotech, TR118200) following the manufacturer’s instructions. Reverse transcription of the RNA samples was carried out for 1 h at 42°C. qPCR was performed using Power SYBR™ Green PCR Master Mix (ThermoFisher Scientific, 4367659) and QuantStudio 3 (Applied Biosystems, A28567). The following primers were used: *GAPDH*: 5′-GGA GCG AGA TCC CTC CAA AAT-3′ (F), 5′-GCT GTT GTC ATA CTT CTC ATG G (R); *AK3*: 5′-AAA CAA CGC CTT ACT GCT C-3′ (F), 5′-TTC CAG CAC CCC TTT TTT C-3′ (R); *CCND1*: 5′-GAT GCC AAC CTC CTC AAC GA-3′ (F), 5′-ACT TCT GTT CCT CGC AGA CC-3′ (R); *JUN*: 5′-GTG CCG AAA AAG GAA GCT GG-3′ (F), 5′-CTG CGT TAG CAT GAG TTG GC-3′ (R); *TCF7*: 5′-GCT GCC ATC AAC CAG ATC CT-3′ (F), 5′-CCT CCT GTG GTG GAT TCT TGG-3′ (R); *TCF7L1*: 5′-CCC CCG CAT TTA AAG GGA CT-3′ (F), 5′-TGT GAA GGG ATG ATC GCC AC-3′ (R); *STK11*: 5′-CGG ACA GGT CCC AGA AGA GG-3′ (F), TCT GTG CCG TTC ATA CAC ACG-3′ (R).

### Subcellular fractionation

Cells were harvested using trypsin-EDTA and resuspended in fractionation buffer [20 mM HEPES (pH 7.4), 10 mM KCl, 2 mM MgCl_2_, 1 mM EDTA, 1 mM EGTA, 1 mM DTT, protease inhibitor cocktail]. After 10 min, cells were lysed by passing through a 26-gauge needle 20 times and incubated for 15 min at 4 °C. Samples were then centrifuged (720 g, 4 °C, 5 min), and pellet was further passed through a 26-gauge needle ten times to lyse remaining intact cells. After centrifugation (720 g, 4 °C, 5 min), pellet contained the nucleus. Meanwhile, the supernatant from the first centrifugation was further centrifuged (10,000 g, 4 °C, 5 min). Pellet contained mitochondria, and supernatant contained cytoplasmic fraction. Pellets from each step were washed and lysed in RIPA buffer. Equal amounts of protein were loaded into SDS-PAGE and analyzed by western blotting. Indicated proteins were used as markers to confirm the identity of each fraction.

### Immunofluorescence and confocal- and super-resolution-microscopy

Cells were seeded on the coverslips and fixed with fresh cold 4% paraformaldehyde solution in PBS for 10 min. Cells were then permeabilized using 0.05 - 0.1% (v/v) Triton X-100 in PBS at room temperature (RT) for 7 min, followed by blocking with tween-20 in PBS (PBST) containing 3% BSA for 1 h at RT. Primary antibodies were diluted in 3% BSA-PBST and incubated with cells overnight at 4°C. Following products were used for the primary antibodies; anti-TOM20 (SCBT, sc-17764, RRID: AB_628381), HA (SCBT, sc-7392), β-catenin (CST, 8480, RRID: AB_11127855), V5 (Abcam, ab27671, RRID: AB_471093). Cells were washed with PBST and further incubated with fluorophore conjugated secondary antibodies (1:200 - 1:1,000 dilution; ThermoFisher Scientific; goat anti-mouse IgG Alexa 488, A32723, RRID: AB_2633275; goat anti-rabbit IgG Alexa 488, A32731; goat anti-mouse IgG Alexa 594, A32742; goat anti-rabbit IgG Alexa 594, A32740; streptavidin Alexa 568, S11226A) for 1 h at RT. Multiple secondary IgGs were incubated sequentially. In particular, secondary IgG against anti-mitochondrial protein IgG was incubated first to prevent dual-staining by anti-rabbit IgG and anti-mouse IgG, which could result in misleading interpretation of fluorescent signals for β-catenin. Nuclear staining was performed with Hoechst 33342 (1:10,000; ThermoFisher Scientific, 62249) for 5 min at RT, and mounted (Sigma, F4680) overnight at 4 °C. Zeiss LSM 700 confocal laser scanning microscope and LSM 980 with Airyscan 2 super-resolution microscope were used to acquire images. Z-stack images were captured at 0.6 µm intervals.

### Quantification of the images

Co-localization between β-catenin and mitochondria or β-catenin and nucleus was analyzed using the BIOP JACoP plugin in ImageJ (RRID: SCR_003070). A single cell boundary was designated, and Pearson’s coefficients (R) between the two fluorescent signals within that area were analyzed. The percentage of co-localization was calculated as follows: (Percentage of β-catenin in cells) = $\frac{R+1}{2}\times 100$ (%). Mitochondrial morphologies were quantified using the Mitochondria Analyzer (2D) plugin in ImageJ. Mean values for individual cells were statistically analyzed.

### Measurement of mitochondrial membrane potential

Flow cytometry (BD Biosciences, FACSCanto II) was used to assess MMP in live cells. Cells were stained with 50 nM MitoTracker Red CMXRos (ThermoFisher Scientific, M7512) for 30 min, detached using T/E, washed, and analyzed. For AK3 KO cells, 50 nM MitoTracker Green FM was used for co-staining, and the fluorescent intensity of individual cells stained with MitoTracker Red CMXRos was compensated using the signals from MitoTracker Green FM to reduce the effects of differences in mitochondrial mass across stable cell lines. Proportion of cell population in each group with high fluorescent intensity was compared and statistically analyzed.

### Mitochondrial ATP measurement

The 3×10³ cells were seeded into an opaque-walled 96-well plate and incubated for 48 h or transfected with the AK3 (RRID: Addgene_37539) plasmid for 24 h. Cells were further incubated for 2 h at 37 °C with either glucose-free media containing 5 mM 2-DG (Merck, 25972) or with media containing high glucose. Cells were lysed and ATP levels were assessed after 10 min following the manufacturer’s instructions (CellTiter-Glo® 2.0, Promega, G9241). Tristar 5 (Berthold) was used to measure luminescent signals. Ratio of ATP^OxPhos^ (2-DG-treated) to ATP^Total^ (untreated) was calculated and statistically analyzed.

### Proteinase K assay

Submitochondrial localization of β-catenin was determined using a proteinase K assay. Mitochondria were purified as described in ‘Subcellular fractionation’ section, and the purified mitochondria were incubated with proteinase K (100 µg/ml) for 30 min at 4 °C. To inactivate proteinase K, 2 mM PMSF was added, and the sample was centrifuged (10,000 g, 4 °C, 5 min). The pellet was lysed in RIPA lysis buffer, immediately loaded onto SDS-PAGE, and analyzed by western blotting.

### Immunoprecipitation assay

Cells were resuspended in IP lysis buffer (10 mM Tris-Cl, pH 7.4; 30 mM NaCl; 1% Triton X-100), sonicated, and incubated for 1 h at 4°C. Samples were then centrifuged (12,000 rpm, 4°C, 5 min) and supernatant was incubated sequentially with the indicated antibody and Protein G-beads (Merck, GE17-0618-05), each for 1 h. Protein G-bound proteins were washed three times and analyzed by western blotting. To identify proteins interacting with mitochondrial β-catenin, mitochondria were purified as described in the ‘Subcellular fractionation’ section, and the purified mitochondria were used for the IP assay.

### Protein stability of β-catenin

Cells were treated with cycloheximide (CHX, 50 μg/mL) for 48 h. Both cells and media were collected, centrifuged, lysed in RIPA lysis buffer, and analyzed by western blotting.

### Proximity labeling assay

Cells were transfected with V5-TurboID-MFN1 and V5-TurboID-MFN2 plasmids for 24 h and treated with mitochondrial inhibitors. Biotin (50 μM) was then added to the cells and incubated for 1 h. Cells were washed three times with PBS, detached using trypsin-EDTA, lysed in RIPA lysis buffer, sonicated, and incubated for 30 min at 4 °C. Lysates were centrifuged (12,000 rpm, 10 min, 4 °C) and supernatants were incubated with streptavidin-agarose conjugate (Millipore, 16-126) for 1 h at 4°C. Streptavidin beads were washed three times with RIPA lysis buffer and eluted with elution buffer (10 mM biotin, 20 mM DTT) by boiling at 95 °C for 10 min. Samples were then analyzed by western blotting.

### Mitochondrial transplantation

Mitochondria were purified from 100 pi scale of HEK293T cells using DMEM instead of fractionation buffer to sustain mitochondrial activity. The 100pi scale of HCT116 were detached using trypsin-EDTA, mixed with the purified mitochondria in 0.5 ml RPMI, and centrifuged (6,000 g, 15 min, RT). Cells were then resuspended, counted, and seeded for the assays. To confirm the mitochondrial transplantation, HEK293T (RRID: CVCL_0063) cells were stained with 50 nM MitoTracker Red CMXRos for 30 min and purified. After transplantation, recipient HCT116 (RRID: CVCL_0291) cells were further stained with 50 nM MitoTracker Green FM and observed under a fluorescent microscope (Olympus, IX71).

### Statistical analysis and software

Statistics were calculated using GraphPad Prism (10.0.3, RRID:SCR_002798), R (4.3.3), and RStudio (2023.12.1 + 402). Data were considered significant if *P*  < 0.05. Paired one-way ANOVA followed by dunnett’s multiple comparison tests was used for three or more groups. Two-tailed paired t-test was used for comparison between two groups. Data distribution was assumed to be normal and variance was assumed to be similar between the groups. Detailed descriptions of statistical methods for each experiment are provided in the figure legends. No statistical methods were used to predetermine sample size, but sample size was chosen following standard practices commonly reported in the literature for the described experiments, with a minimum of three biological repeats. Images, data, and illustrations were generated using Zen 3.0 (Blue edition), Image J (RRID: SCR_003070, 2.14.0/1.54 f), Adobe Photoshop (RRID: SCR_014199), and BioRender.

### Randomization and blinding

In all cell culture experiments, cell dishes were randomly assigned to different experiments and treatment groups. For the quantification of cell images, individual cells were randomly selected and analyzed without bias. No data were excluded from all analyses in this study, except in cases of experimental failure. Blinding was not applied, since none of the analyses in this study relies on the subjective perspective of the investigator.

## Supplementary information

Reproducibility Checklist

Supplementary figure legends

Supplementary figure 1

Supplementary figure 2

Supplementary figure 3

Supplementary figure 4

Supplementary figure 5

Supplementary figure 6

Supplementary figure 7

Uncropped blot images

## Data Availability

Transcriptomic datasets from human patients are available at The Cancer Genome Atlas (TCGA) database (http://firebrowse.org) and Gene Expression Omnibus (NCBI-GEO, RRID:SCR_005012, https://www.ncbi.nlm.nih.gov/geo/). All other data are provided in the manuscript or the supplementary information.
