# Supplementary material for: Mitochondrial AK3 inhibits nuclear β-catenin localization and its activation through enhancing mitochondrial activity
Source: Cell Death Dis. 2026 Apr 22;17(1):529. doi: 10.1038/s41419-026-08777-z (PMC13234369; doi:10.1038/s41419-026-08777-z)
Supplement: Supplementary file 2 — Supplementary figure legends [file 41419_2026_8777_MOESM2_ESM.docx]

**Supplementary figure legends**

**figure S1. AK3 is downregulated and correlated with OxPhos pathway in cancer patients.**

**(A and B)** Comparison of AK3 expression in LUAD patient samples. LUAD datasets containing microarray data, including GSE18842 (A) and GSE33356 (B), were analyzed to compare AK3 mRNA levels between tumor tissues and matched non-tumor tissues. Dot plots with mean value (black bar) were statistically analyzed by paired Student’s t-test.

**(C)** TCGA-LUAD dataset from Fig. 1B was sorted by tumor stages and statistically analyzed (one-way ANOVA followed by Dunnett’s test for multiple comparison).

**(D-H)** Comparison of AK3 expression in CRC patient samples. CRC datasets containing microarray data, including TCGA_COAD (D), GSE9348 (E), GSE35279 (F), GSE32323 (G), and GSE110224 (H), were analyzed to compare AK3 mRNA levels between tumor tissues and matched non-tumor tissues. Dot plots with mean value (black bar) were statistically analyzed by paired Student’s t-test.

**(I)** All probes from GSE31210 were analyzed to determine their Pearson correlation coefficients and P values in relation to AK3 expression. The genes correlated with AK3 (R ≥ 0.3 or R ≤ -0.3) were annotated as ‘AK3 gene signature’.

**(J and K)** OxPhos scores and AK3 and AK4 mRNA levels for CRC tumors and non-tumors. Pearson correlation coefficients and P values between OxPhos scores and AK3 (J) or AK4 (K) mRNA levels were statistically analyzed in TCGA_COADREAD. Linear regression lines along with 95% confidence bands (gray) were shown. (*P < 0.05, **P < 0.01, ***P < 0.001, ****P < 0.0001).

**figure S2. AK3 attenuates β-catenin signaling and cell proliferation in A549 and HCT116 cells.**

**(A)** Cell proliferation rates of A549 AK3-KO cells analyzed by MTT assay after 48 h. n = 4.

**(B and C)** Cell proliferation rates of A549 cells expressing exogenous AK3 for 24 h. Cells were analyzed by MTT assay (B) or counting cell numbers for 4 days (C). n = 4 (B) and n = 3 (C).

**(D)** Cell migration rates of A549 cells overexpressing AK3. A549 cells were transfected with AK3 for 24 h followed by wound healing assay. Area of migrated cells after 28 h was quantified and statistically analyzed. Control, n = 40; AK3, n = 38 (left).

**(E)** Immunoprecipitation (IP) assay in A549/Ctrl and A549/sgAK3 cells. The interactions between β-catenin and E-cadherin or LEF1 were analyzed by IP assay. Signals on the blots for anti-β-catenin immunoprecipitates were quantified and are shown below the images.

**(F and G)** Immunoblot analysis of HCT116 AK3-depleted cells (F) and HCT116 cells overexpressing AK3 (G). AK3-depleted cells were generated by two different guide RNAs (#1 or #2). Signals on the blots were quantified and statistically analyzed. n = 3.

**(H and I)** Cell proliferation rates of HCT116 AK3-depleted cells were analyzed by MTT assay (H) or counting cell numbers for 4 days (I). n = 3.

**(J)** Cell proliferation rates of HCT116 cells overexpressing AK3. HCT116 cells were transfected with AK3 for 24 h followed by MTT assay. n = 3.

**(K)** Activation of β-catenin signaling in HCT116 cells ectopically expressing AK3 for 24 h was assessed by TOPflash/FOPflash assay. n = 3.

Bars and values represent mean ± s.e.m. (*P < 0.05 **P < 0.01, ***P < 0.001, ****P < 0.0001). Two-tailed paired t-test (B, C, D, G, J). Paired one-way ANOVA followed by dunnett’s multiple comparison tests (A, F, H, I, K).

**figure S3. AK3 modulates nuclear β-catenin accumulation through its enzymatic activity.**

**(A and B)** Subcellular fractionation assays of HCT116 cells expressing AK3-Flag (A) and A549 AK3-KO cells (B). Markers for subcellular organelles are shown (left). Mito-matrix; mitochondrial matrix, IMM; inner mitochondrial membrane. Signals on the blots were quantified and statistically analyzed. Two-tailed paired t-test. n = 3.

**(C)** Immunostaining of β-catenin and mitochondrial AK4 in A549 cells expressing AK4-HA. Scale bars indicate 10 μm.

**(D)** Immunoblotting showing expression of AK3-∆1 and AK3-∆2 mutants in HEK293T cells.

**(E and F)** Immunostaining of β-catenin and mitochondria in HeLa cells overexpressing AK3 WT or AK3-∆2 mutant (E). Co-localization between β-catenin and mitochondria is presented as the percentage of co-localization (F). Bars represent mean ± s.e.m. One-way ANOVA followed by dunnett’s multiple comparison tests. Scale bars indicate 5 μm.

**(G)** MMP alteration in A549 AK3 KO cells. Cells were treated with 10 μM CCCP for 2 h, stained with Mitotracker Red CMXRos, and then analyzed by flow cytometry. Bars represent mean ± s.e.m. Paired one-way ANOVA followed by dunnett’s multiple comparison tests. n = 3 (*P < 0.05 **P < 0.01, ***P < 0.001, ****P < 0.0001).

**figure S4. Genetic ablation of MFN1 and MFN2 alters β‑catenin subcellular localization and expression levels.**

**(A)** Purified mitochondria from HEK293T cells expressing Flag-CTNNB1 were subjected to immunoprecipitation (IP) assay using Flag antibody.

**(B)** Immunoprecipitation (IP) assay using Flag antibody in HEK293T cells expressing GFP-CTNNB1 and either MFN1-Flag or MFN2-Flag.

**(C)** Purified mitochondria from A549 cells expressing Flag-CTNNB1 were subjected to IP assay using Flag antibody.

**(D)** Mapping MFN1 domain that interacts with β-catenin. HCT116 cells were transfected with MFN1-Flag WT or mutant for 24 h and subjected to IP assay using Flag antibody.

**(E-G)** Immunoblot analysis of β-catenin and mitochondrial proteins in HCT116 MFN1-KO (E), MFN2-KO (F), and double-KO (G) cells generated by different guide RNAs (#1 and 2).

(**H**) Mitochondrial morphology of HCT116 WT and MFN-KO cells was observed under confocal microscope following mitotracker staining. Scale bars indicate 2 μm.

**(I)** Subcellular fractionation assay in HeLa WT and MFN-KO cells. HeLa WT, MFN1-KO, and MFN2-KO cells generated by the different guide RNAs (#1 and #2). Protein markers of the indicated organelles are shown (left).

**(J)** Protein stability of β-catenin in A549 cells overexpressing AK3-Flag. After transfection with AK3-Flag for 24 h, cells were incubated with 100 μM cycloheximide (CHX) for 24 h or 48 h, and analyzed by western blot assay. Signals on the blots were quantified and statistically analyzed. Paired two-way ANOVA followed by dunnett’s multiple comparison tests. n = 3 (*P < 0.05 **P < 0.01, ***P < 0.001, ****P < 0.0001).

**(K and L)** Pearson’s coefficients between MFN and other genes in GSE31210. MFN1 and MFN2 gene signatures were annotated by calculating Pearson’s correlation coefficients. Gene ontology analysis of MFN1 (K) and MFN2 (L) gene signatures was performed (Fisher exact test).

**figure S5. Subcellular distribution of β‑catenin mutants harboring alterations at PTM sites.**

**(A)** Subcellular fractionation assay to analyze localization of Flag-β-catenin and β-catenin-Flag in HEK293T cells.

**(B)** Immunoprecipitation (IP) assay showing the interaction between β-catenin ∆2 and mitofusins. Purified mitochondria from HEK293T cells expressing Flag-CTNNB1 ∆2 were subjected to IP assay using Flag antibody.

**(C and D)** Subcellular fractionation assay to examine localization of β-catenin PTM mutants harboring a mutation at the reported residues. The roles of β-catenin associated with its PTM are indicated at the top. The representative immunoblots are full data of Fig. 5C (C). Signals on the blots were quantified and statistically analyzed (D). Paired one-way ANOVA followed by dunnett’s multiple comparison tests. n = 3 (*P < 0.05 **P < 0.01, ***P < 0.001, ****P < 0.0001).

**figure S6. Validation of TurboID-MFN proximity labeling assay.**

**(A)** Fluorescent images showing mitochondrial localization of V5-TurboID-MFN1 and V5-TurboID-MFN2. Scale bars; 10 μm.

**(B and C)** Streptavidin-pulldown assay in HeLa cells expressing V5-TurboID-MFN1 (B) or V5-TurboID-MFN2 (C) and exposed to 50 μM biotin for 3 h. Biotinylated proteins were analyzed by western blotting.

**(D)** Visualization of biotinylated proteins by streptavidin-staining. HeLa cells expressing V5-TurboID-MFN2 were incubated with 50 μM biotin for 1 h and stained with anti-V5-tag antibody and streptavidin. Scale bars; 10 μm.

**(E)** Mitochondrial localization of V5-TurboID-MFN-2S mutants. HeLa cells were transfected with V5-TurboID-MFN1-2S or V5-TurboID-MFN2-2S mutants for 24 h and co-stained with mitotracker and anti-V5-tag antibody. Scale bars; 10 μm.

**(F and G)** Mitochondrial ROS levels in A549 cells. Mitochondrial superoxide levels were measured by MitoSOX staining in A549/Ctrl and A549/sgAK3 cells (F) or A549/Ctrl cells and A549/AK3 cells (G). Cells were pre-treated with 10 μM CCCP or 5 μg/ml rotenone for 2 h, or transfected with AK3 for 24 h. One-way ANOVA followed by dunnett’s multiple comparison tests. Bars represent mean ± s.e.m. n = 3.

**(H)** DCP-bio1 assay in A549 cells. Cells lysate in pH 7.0 or pH 8.0 lysis buffer were pulled-down with streptavidin. DCP-bio1-bound sulfenylated proteins were analyzed by immunoblotting.

**(I)** Hypothetical model showing the AK3-mediated MFN dimerization to promote the MFN–β-catenin interaction. AK3-enriched mitochondria exhibit elevated MMP, reflecting an increase in proton-motive force and acidification of the IMS. Enhanced OxPhos increases ATP production and mitochondrial ROS levels. Together, these acidic condition in the IMS and ROS abundance in the mitochondria might promote sulfenylation of the MFN cysteine residues, leading to MFN dimer formation and strengthening the MFN–β-catenin interactions.

**figure S7. Transplantation of mitochondria into HCT116 cells attenuates cell proliferation and β-catenin signaling.**

**(A)** Transplanted mitochondria stained with Mitotracker Red were observed under a fluorescent microscope. All mitochondria, including both donor and recipient mitochondria, were also stained with Mitotracker Green. Scale bars; 5 μm.

**(B)** Cell proliferation rates of mitochondria-transplanted HCT116 cells. Cell numbers were counted at day 4 and represented as values with mean ± s.e.m. (n = 3; two-tailed paired t-test).

**(C)** TOPflash/FOPflash assay in the recipient HCT116 cells. Bars represent mean ± s.e.m. (n = 5; two-tailed paired t-test).

**(D)** β-catenin signaling proteins and mitochondrial proteins in the mitochondria-transplanted HCT116 cells. Two distinct mitochondrial TOM20 proteins were detected after mitochondrial transplantation (endogenous TOM20 from HCT116 cells; exogenous TOM20 from HEK293T cells). Signals on the blots were quantified and statistically analyzed. (n = 3; two-tailed paired t-test). (*P < 0.05 **P < 0.01, ***P < 0.001, ****P < 0.0001).
