# Supplementary figures and images for: Mitochondrial AK3 inhibits nuclear β-catenin localization and its activation through enhancing mitochondrial activity
Source: Cell Death Dis. 2026 Apr 22;17(1):529. doi: 10.1038/s41419-026-08777-z (PMC13234369; doi:10.1038/s41419-026-08777-z)

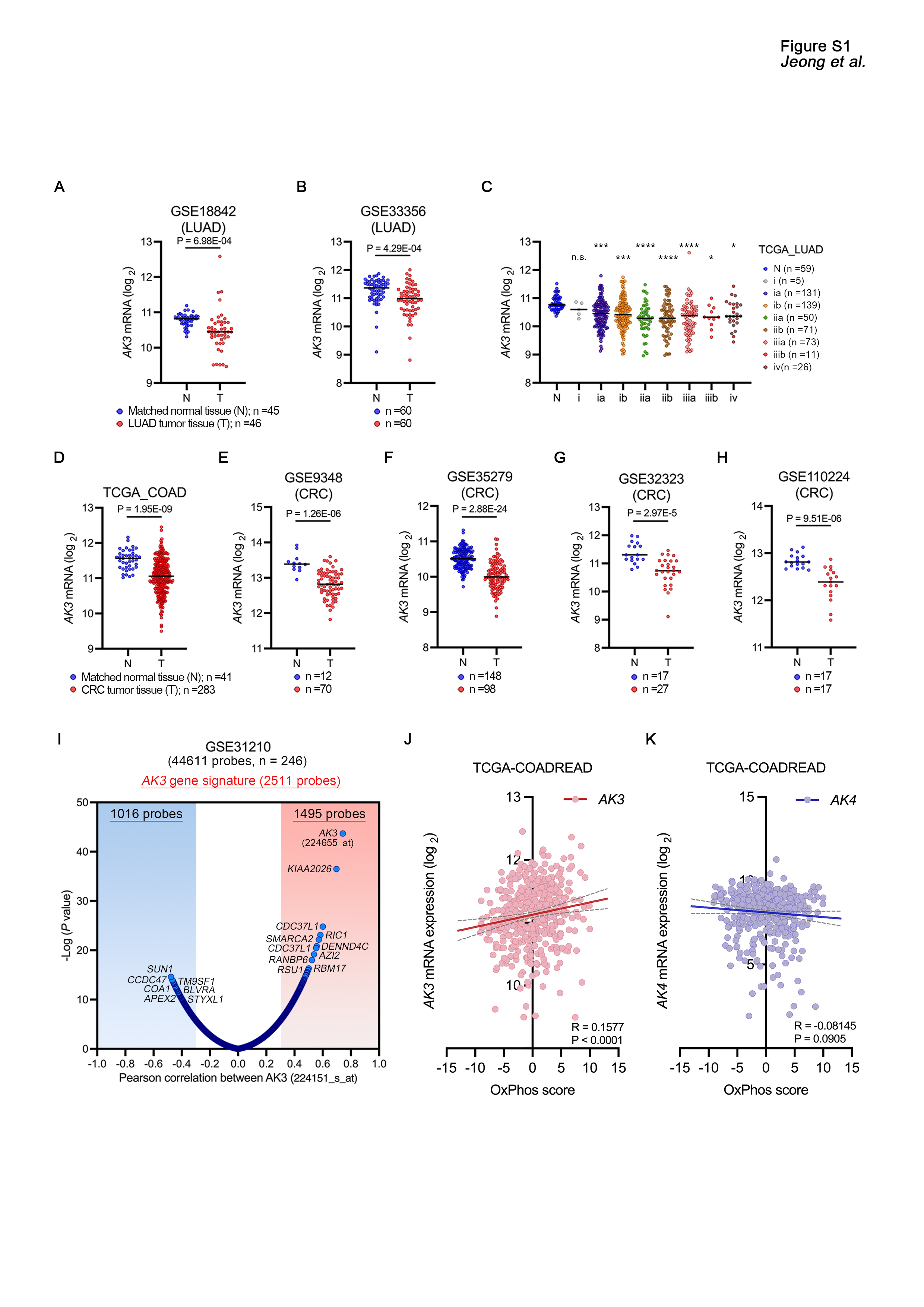

Supplement: Supplementary file 3 — Supplementary figure 1 [file 41419_2026_8777_MOESM3_ESM.jpg]

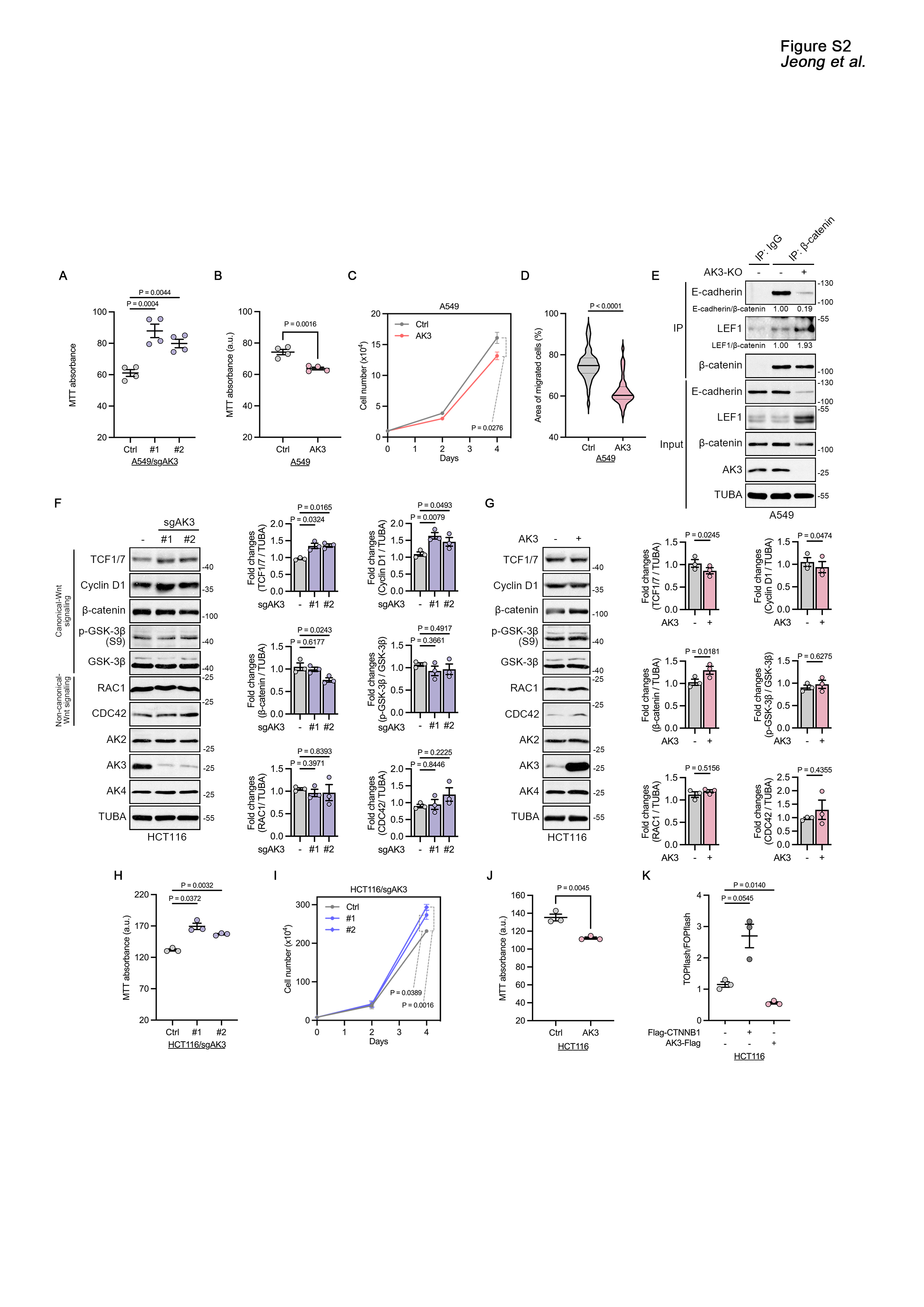

Supplement: Supplementary file 4 — Supplementary figure 2 [file 41419_2026_8777_MOESM4_ESM.jpg]

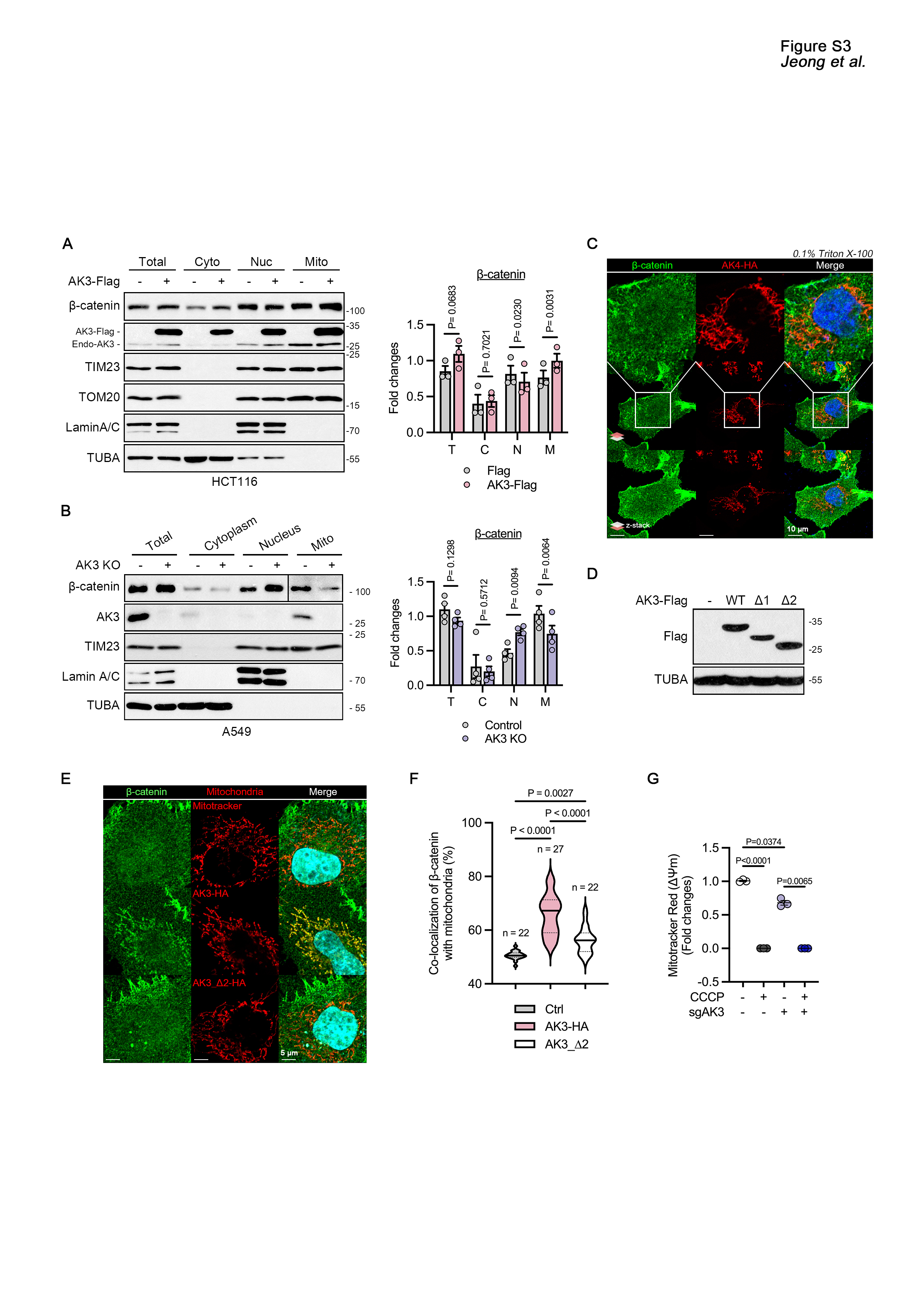

Supplement: Supplementary file 5 — Supplementary figure 3 [file 41419_2026_8777_MOESM5_ESM.jpg]

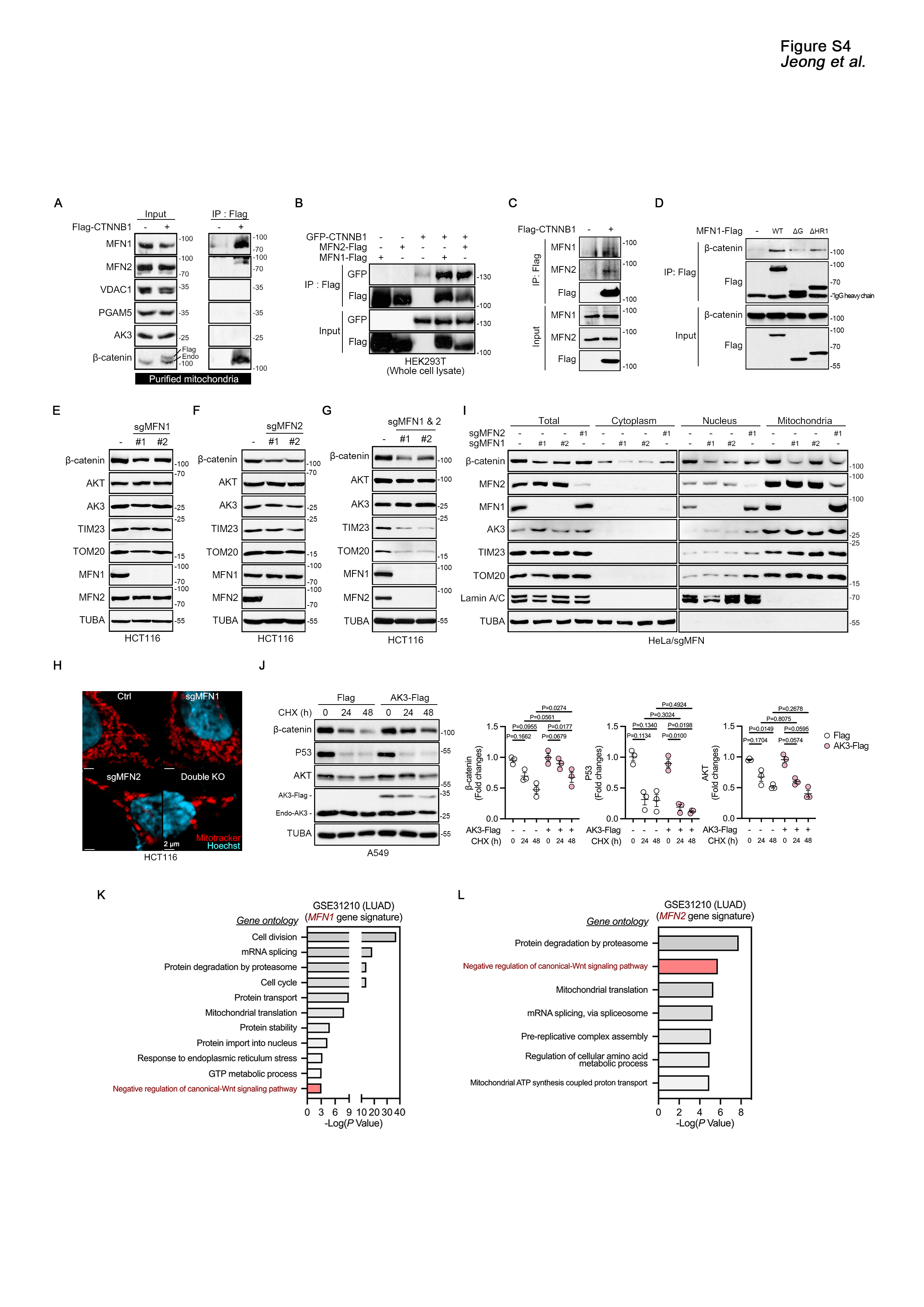

Supplement: Supplementary file 6 — Supplementary figure 4 [file 41419_2026_8777_MOESM6_ESM.jpg]

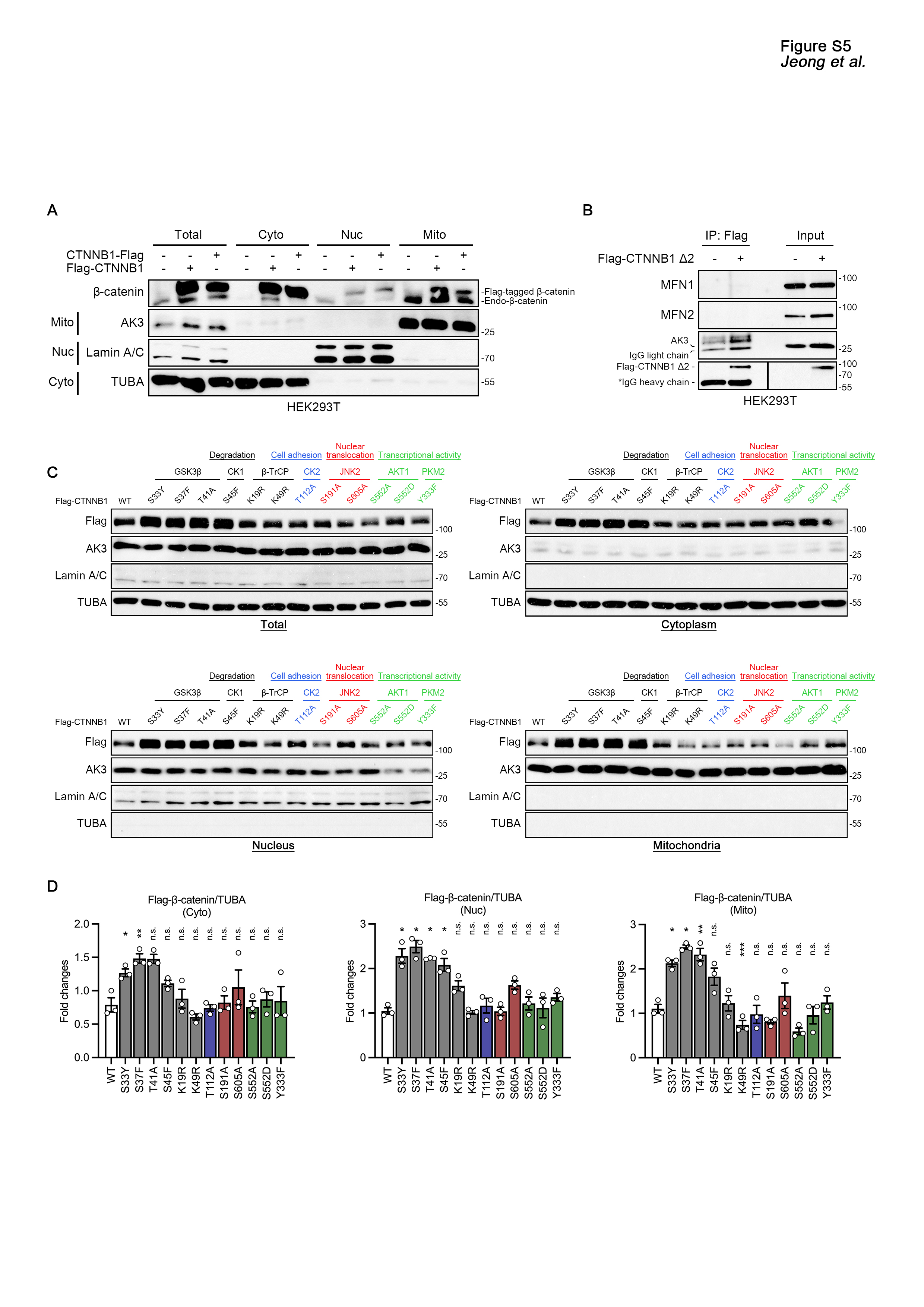

Supplement: Supplementary file 7 — Supplementary figure 5 [file 41419_2026_8777_MOESM7_ESM.jpg]

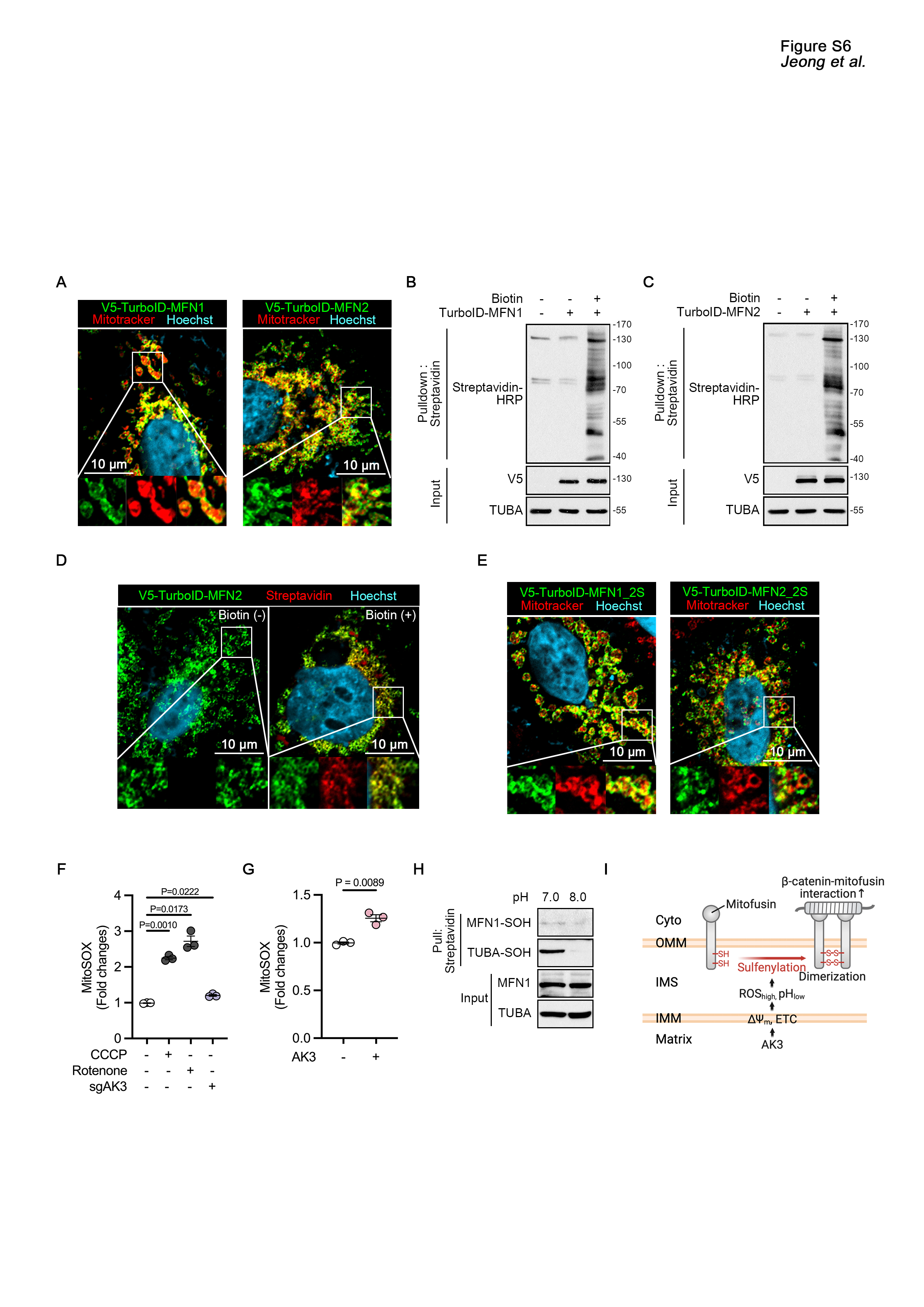

Supplement: Supplementary file 8 — Supplementary figure 6 [file 41419_2026_8777_MOESM8_ESM.jpg]

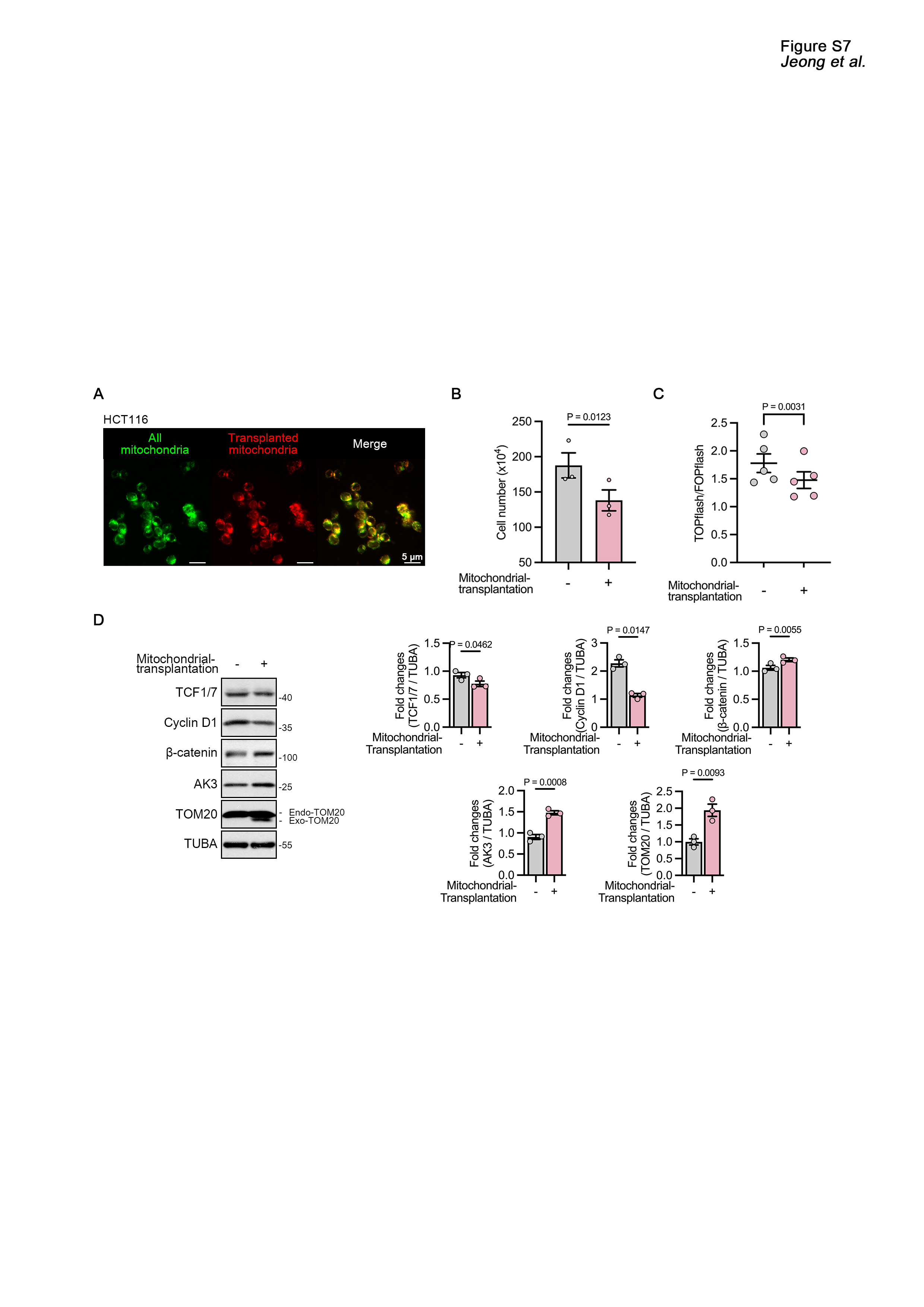

Supplement: Supplementary file 9 — Supplementary figure 7 [file 41419_2026_8777_MOESM9_ESM.jpg]
