# Supplementary material for: Mitochondrial AK3 inhibits nuclear β-catenin localization and its activation through enhancing mitochondrial activity
Source: Cell Death Dis. 2026 Apr 22;17(1):529. doi: 10.1038/s41419-026-08777-z (PMC13234369; doi:10.1038/s41419-026-08777-z)

Figure 2A

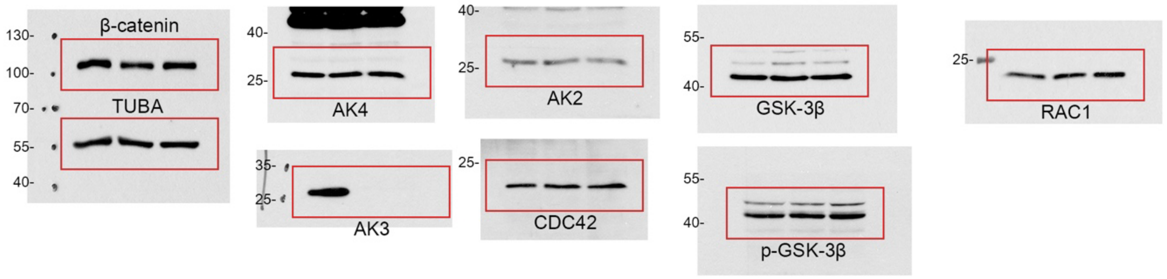

Figure 2B

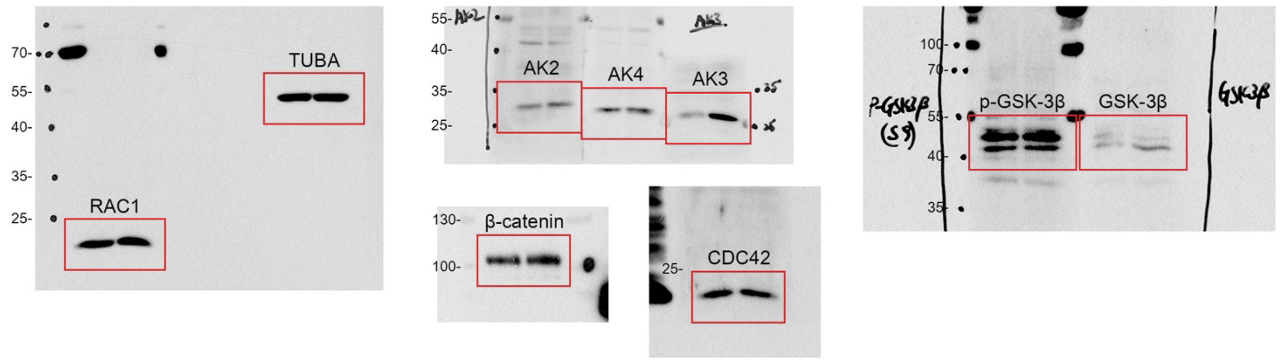

Figure 3A

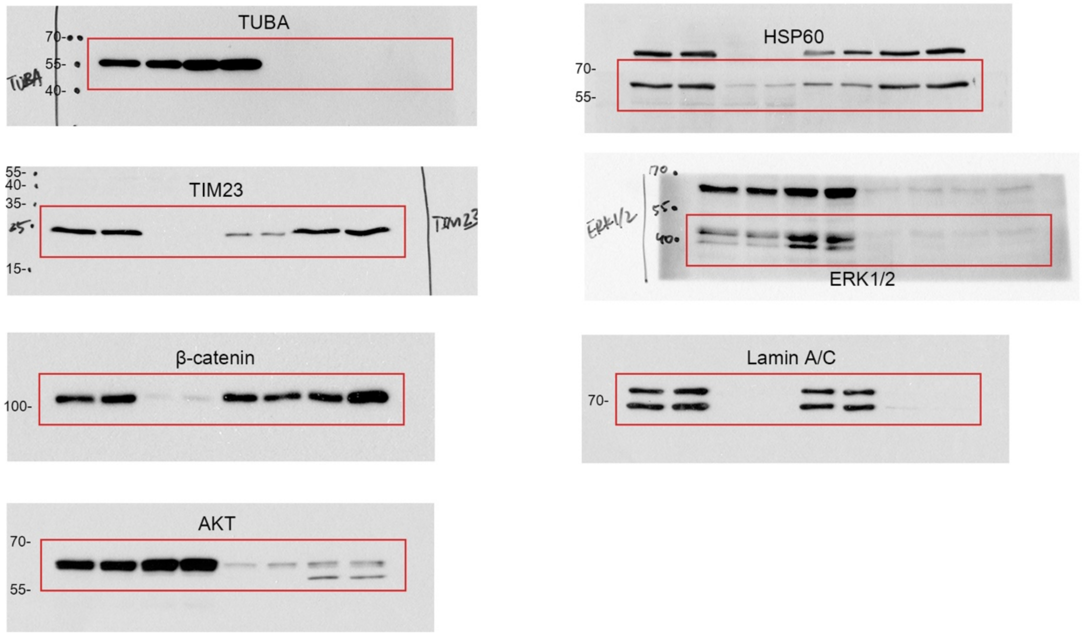

Figure 4A

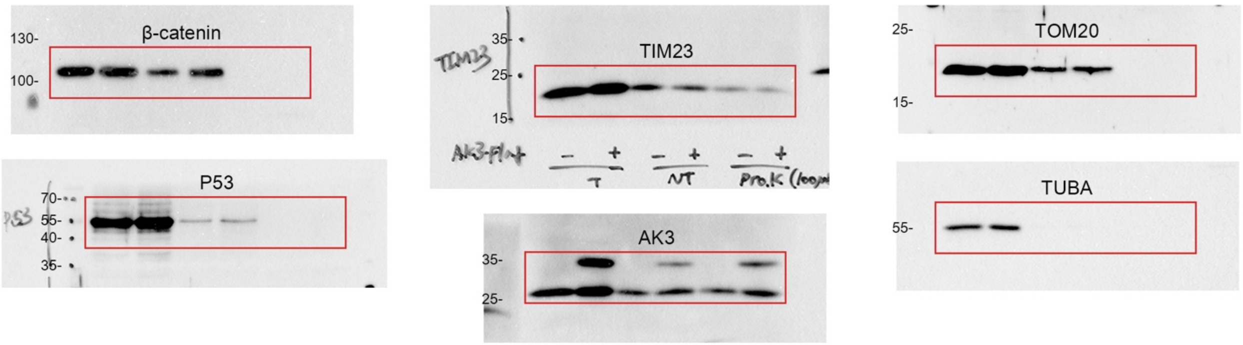

Figure 4B

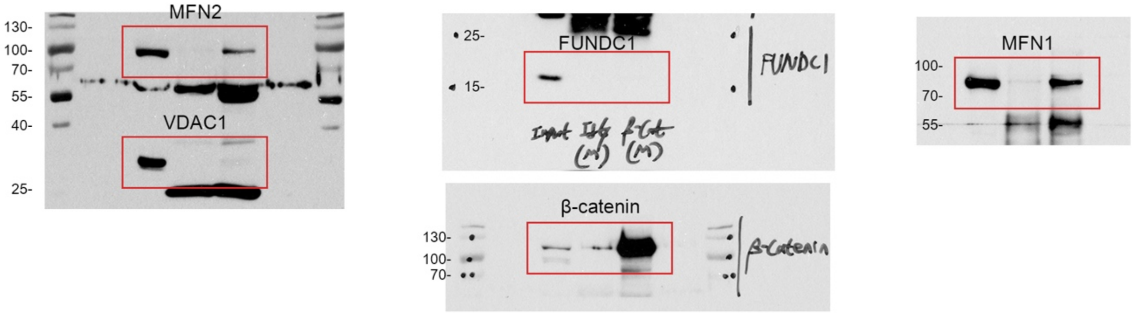

Figure 4C

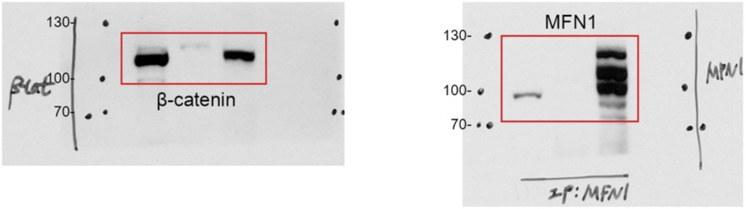

Figure 4D

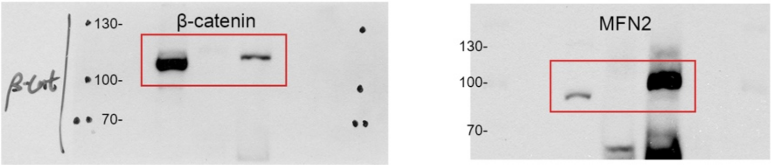

Figure 4F

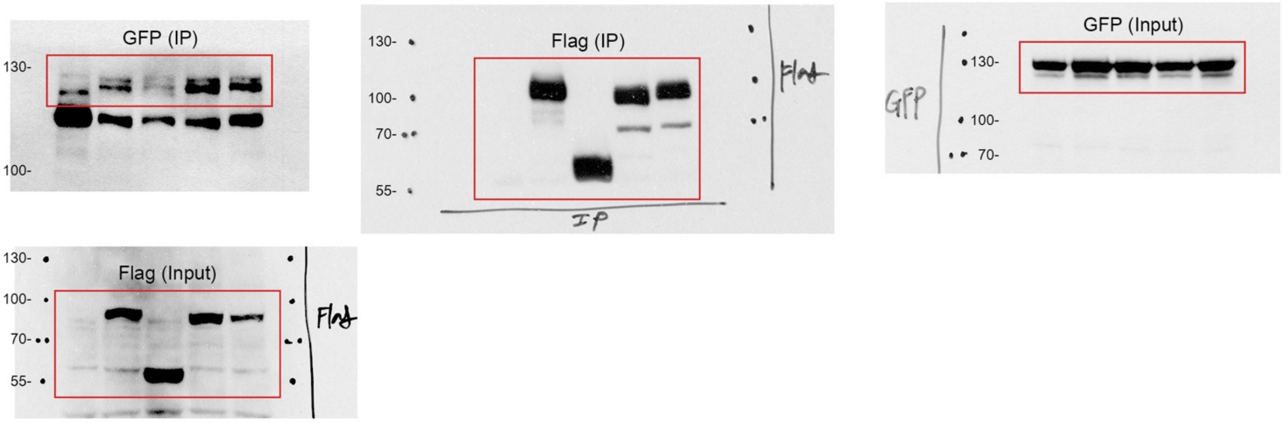

Figure 4G

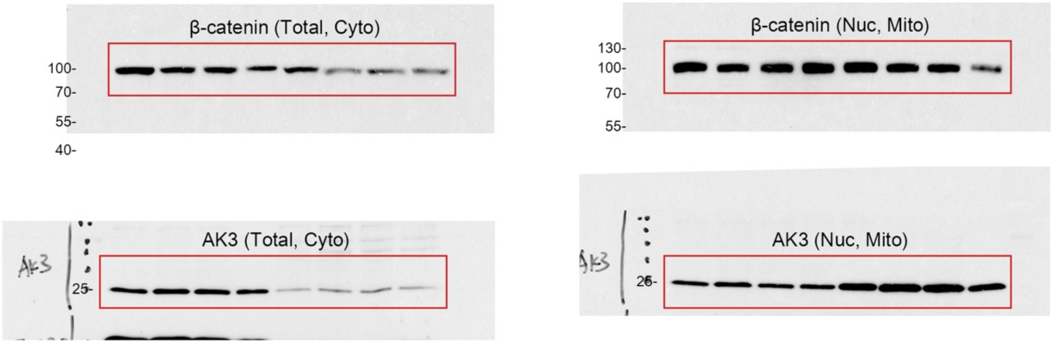

Figure 4G

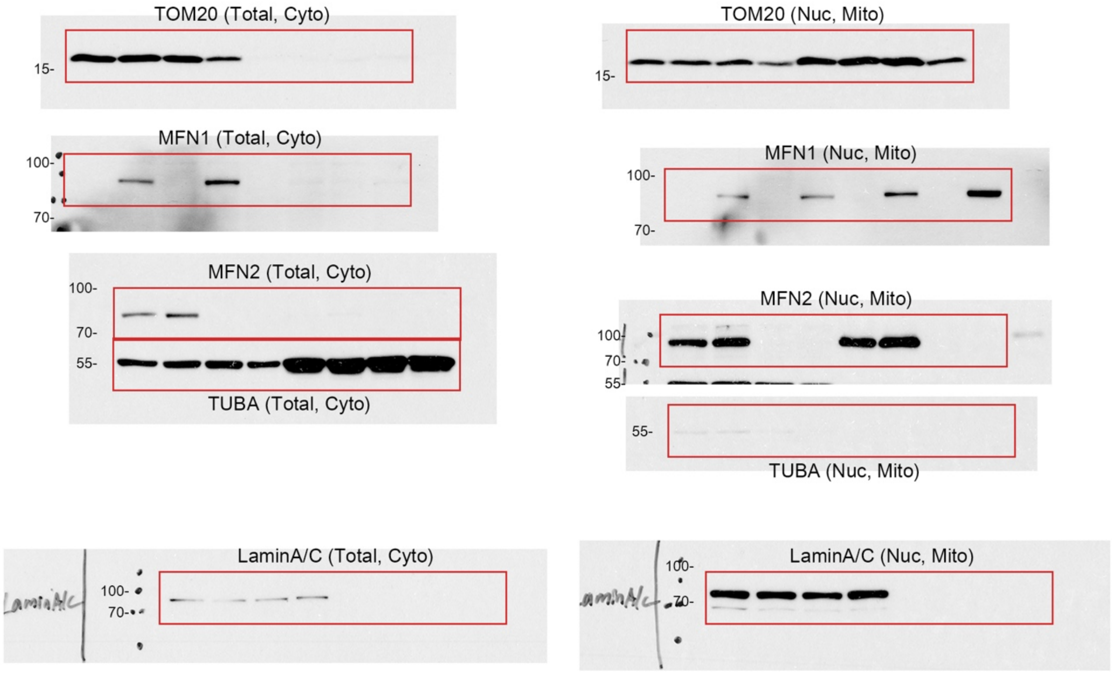

Figure 5B

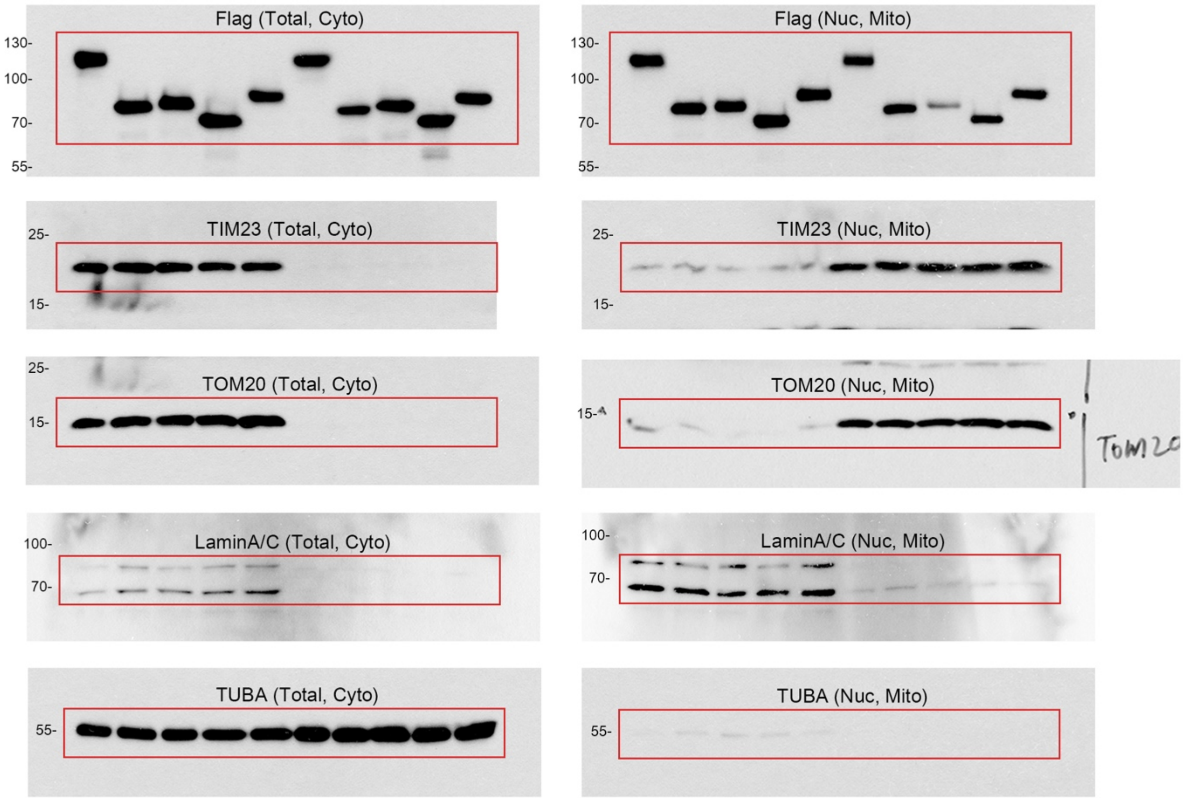

Figure 5C

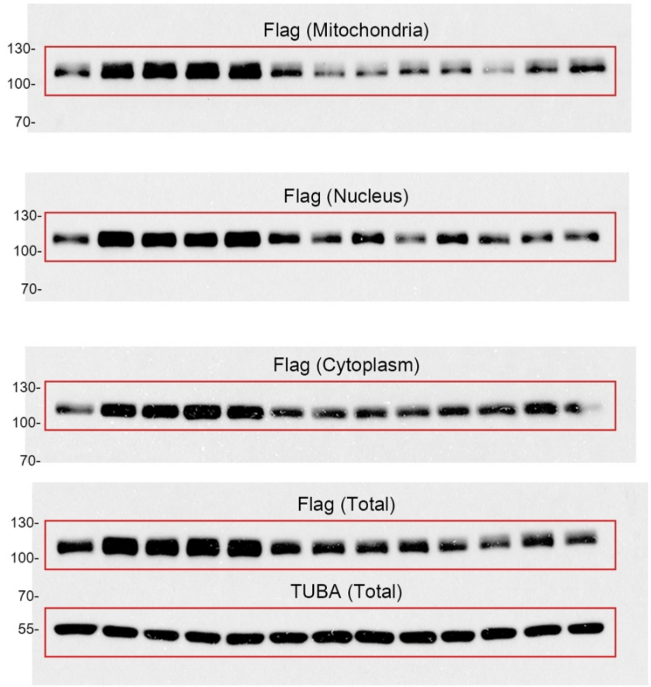

Figure 5G

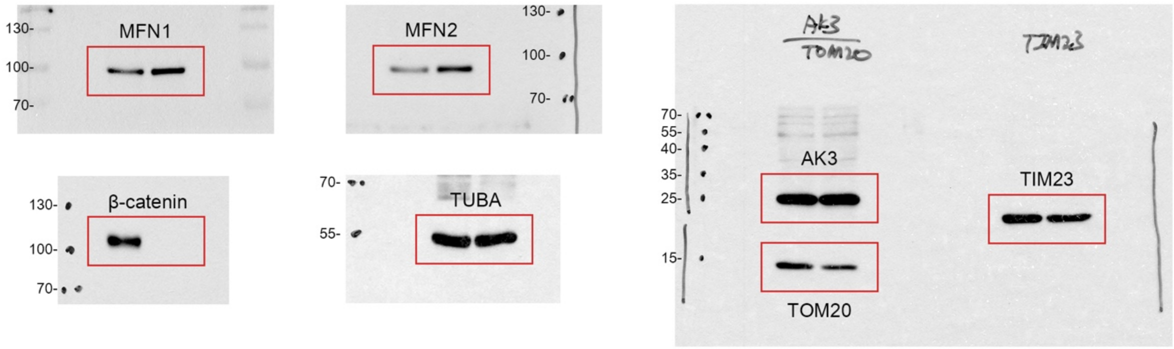

Figure 6B

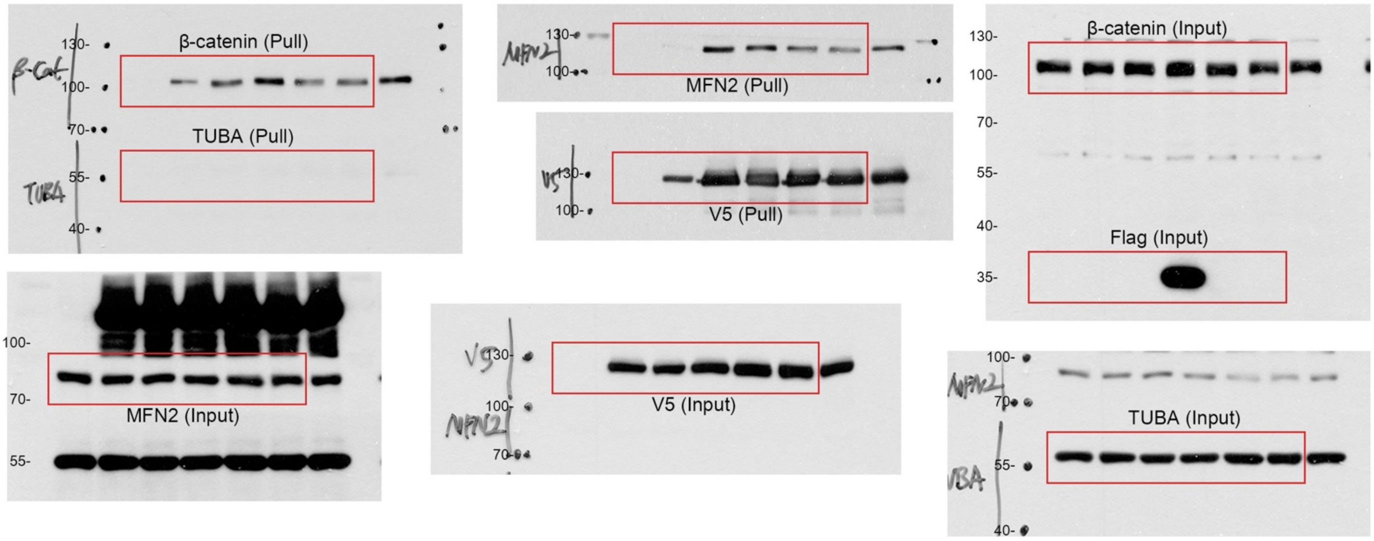

Figure 6C

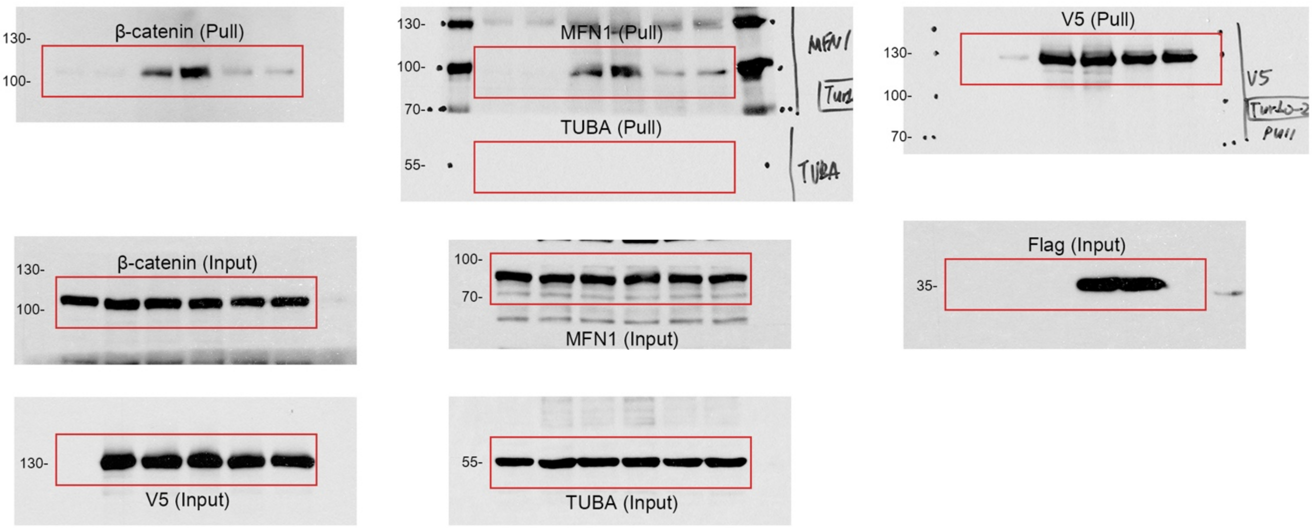

Figure 6D

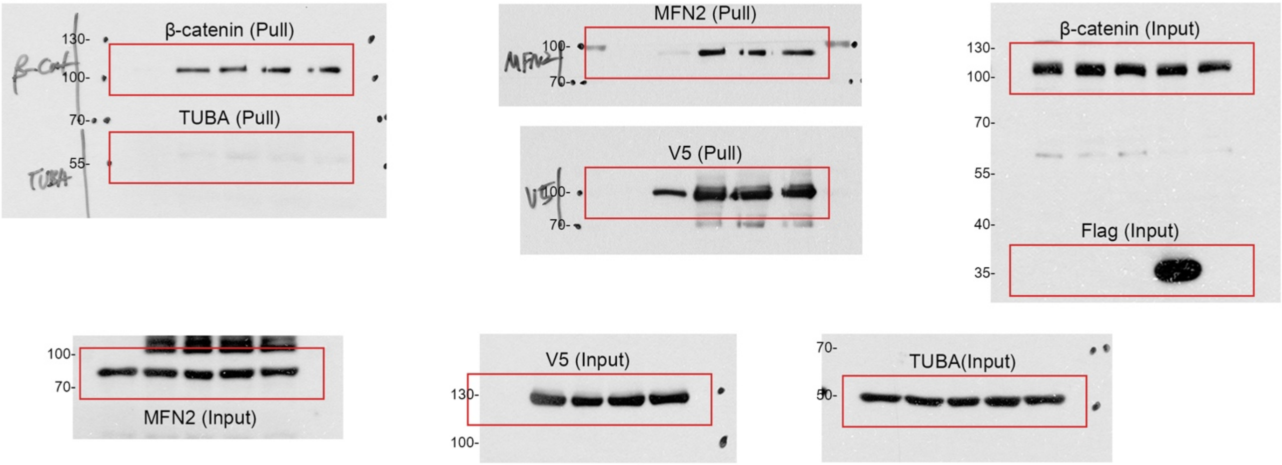

Figure 6E

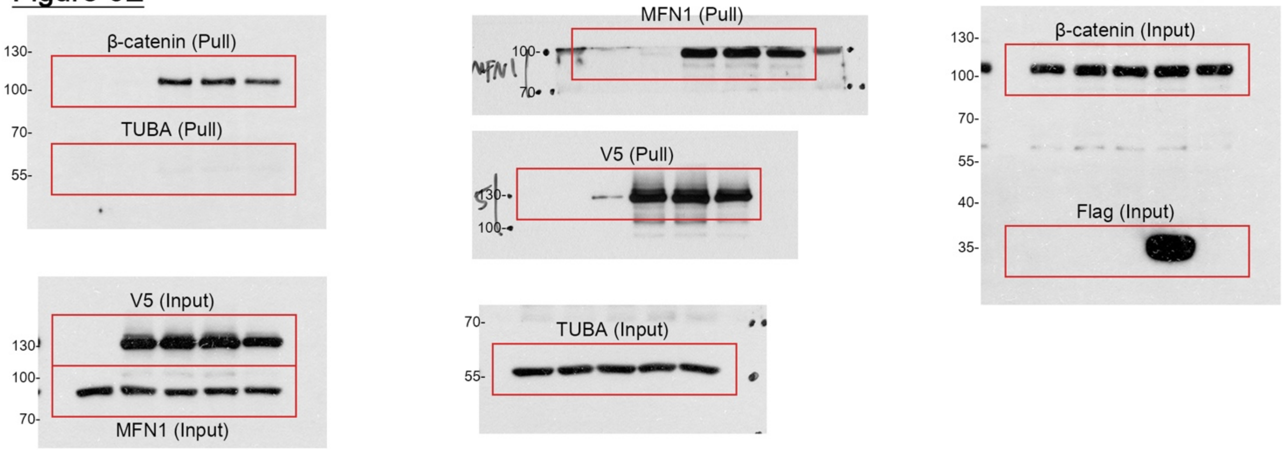

Figure 6F

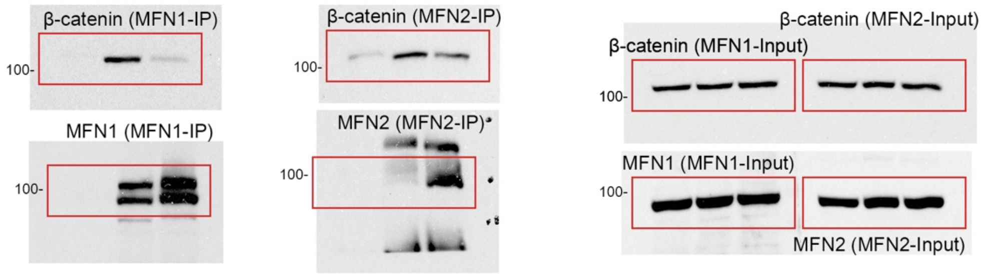

Figure 6G

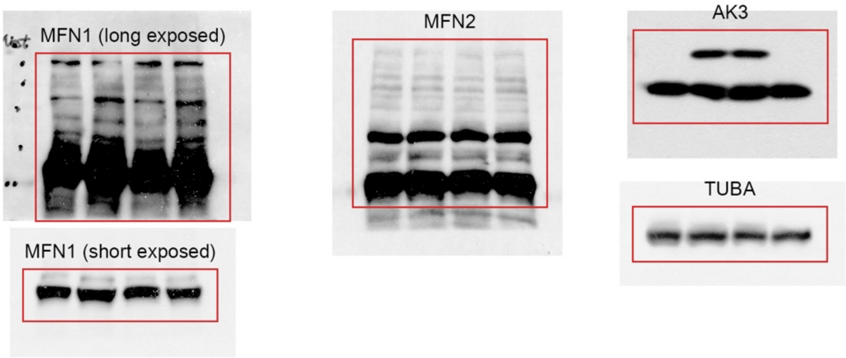

Supplement: Supplementary file 10 — Uncropped blot images [file 41419_2026_8777_MOESM10_ESM.pdf]
